# Beverage consumption on bone and joint disorders: an umbrella review

**DOI:** 10.7189/jogh.15.04222

**Published:** 2025-08-04

**Authors:** Han Tan, Peiyuan Tang, Hua Chai, Wenbo Ma, Yangbin Cao, Bin Lin, Ying Zhu, Wenfeng Xiao, Ting Wen, Bangbao Lu, Yusheng Li

**Affiliations:** 1 Department of Orthopedics, Xiangya Hospital, Central South University, Hunan, China; 2 National Clinical Research Center for Geriatric Disorders, Xiangya Hospital, Central South University, Changsha, China; 3 Xiangya School of Medicine, Central South University, Changsha, China

## Abstract

**Background:**

Bone and joint disorders significantly contribute to disability worldwide. While meta-analyses have explored the relationship of these diseases and beverage consumption, the results remain inconsistent. In this umbrella review, we synthesised existing evidence to clarify these associations.

**Methods:**

We conducted a systematic search in PubMed, Embase, Cochrane, and Web of Science up to January 2025. Two independent reviewers screened studies and extracted data based on predefined criteria. We included meta-analyses and systematic review studies. We used the Graphical Representation of Overlap for Overviews tool to manage overlapping studies and assessed methodological quality and evidence levels using AMSTAR 2 tool and the GRADE system. We narratively synthesised the findings and summarised them in tables.

**Results:**

We included 20 meta-analyses. Tea consumption was associated with a reduced risk of osteoporosis (odds ratio (OR) = 0.71; 95% confidence interval (CI) = 0.60, 0.83; *P* < 0.0001; *I*^2^ = 13%), while coffee showed mixed results. Alcohol intake increased osteoporosis risk (OR = 2.95; 95% CI = 1.78, 4.9; *P* < 0.0001; *I*^2^ = 0%). Sugar-sweetened beverages raised gout risk (relative risk (RR) = 1.35; 95% CI = 1.18, 1.55, *P* < 0.05; *I*^2^ = 40.1%) and serum uric acid levels, while coffee lowered gout risk (RR = 0.43; 95% CI = 0.31, 0.59; *P* < 0.001; *I*^2^ = 0%). Coffee was associated with a higher risk of rheumatoid arthritis (RR = 1.3; 95% CI = 1.04, 1.62; *P* < 0.05; *I*^2^ = 0%), while tea showed no significant effect.

**Conclusions:**

Tea may benefit bone and joint health, while alcohol and sugar-sweetened beverages are associated with a higher incidence of bone and joint conditions, such as osteoporosis, rheumatoid arthritis, and gout. These findings emphasise the importance of dietary choices in preventing bone and joint disorders.

**Registration:**

PROSPERO (CRD42024551504).

Bone and joint disorders (BJDs) encompass a variety of conditions that commonly affect joint function, including osteoarthritis, rheumatoid arthritis (RA), gout, and osteoporosis [1]. These chronic conditions predominantly impact the elderly population and are recognised as leading causes of disability globally [2,3]. Affected individuals often experience chronic joint pain, limited joint mobility, and reduced quality of life [4]. The long-term medical expenses associated with managing these disorders impose a significant burden on both individuals and healthcare systems [5]. Therefore, identifying modifiable risk factors that contribute to the onset and progression of BJDs is of considerable public health importance.

Beverages constitute a significant portion of our daily dietary intake. Coffee, for example, is the second most popular beverage globally, with trade volumes exceeding USD 10 billion [6]. An umbrella review reported that coffee consumption is associated with an increased risk of RA in women [7]. Tea, the third most popular beverage globally, demonstrated beneficial effects in the prevention of the development of cardiovascular diseases and cancer when consumed in moderate amounts (2–3 cups per day) [8] and has been found to reduce the risk of osteoporosis and RA [9,10]. Research on SSBs, which includes fruit juices, carbonated and noncarbonated soft drinks, and sports and energy drinks [11], has mainly focussed on their impact on BJDs. Findings from other studies have shown that higher SSB consumption is associated with an increased risk of gout [12–14]. Alcohol consumption – especially in excessive amounts – has been linked to various adverse effects on bone health and increased risk of osteoporosis [15,16].

Although many meta-analyses (MAs) of randomised controlled trials (RCTs), case-control studies, cohort, and cross-sectional studies have focussed on the association between diverse beverage consumption and various BJDs, their results have been inconsistent. Therefore, we aimed to clarify the relationship between beverage consumption and BJDs through this umbrella review, hypothesising that tea and coffee may decrease the incidence of BJDs, such as gout, osteoporosis, and RA, whereas SSB consumption and alcohol may increase the risk of BJDs.

## METHODS

An umbrella review synthesises data from multiple MAs across various outcomes [17]. We adopted this methodological approach for our study, registering it in PROSPERO (CRD42024551504) and designing it per following the procedures outlined in the Cochrane Handbook [18,19]. We complied with the PRIOR guidelines in reporting our findings (Table S1 in the **Online Supplementary Document**).

### Search methodology

We searched four databases – PubMed, Embase, Cochrane, and Web of Science – up to January 2025 by combining subject terms and free-text keywords, such as ‘gout’, ‘osteoarthritis’, ‘osteoporosis’, ‘rheumatoid arthritis’, ‘coffee’, ‘tea’, ‘alcoholic beverage’, and ‘sugar-sweetened beverages’ (Text S1 in the **Online Supplementary Document**).

### Study selection

We used the PICOS question as the basic eligibility criteria for the umbrella review:

− P: Patients who are with BJDs;
− I: Patients who have beverage consumption (coffee, tea, SSBs, and alcohol);
− C: Patients who don’t consume beverage or with the minimum concentration;
− O: the incidence of gout, RA, and osteoporosis;
− S: MA and systematic review.

We therefore included systematic reviews and MAs assessing the impact of beverage consumption (coffee, tea, SSBs, and alcohol) on BJDs (gout, RA, and osteoporosis). Two researchers (HT and PT) independently screened the literature based on predefined inclusion and exclusion criteria.

We excluded MAs of Mendelian randomisation studies, because their methodological framework differs from conventional observational MAs, as they focus on genetic proxies rather than direct associations between exposure and outcome. We also excluded studies with insufficient data availability, non-English studies, as well as animal and cell studies.

### Overlapping discovery and processing

If two or more MAs assessed the same outcome, we assumed there may be overlap in the original studies. To visually identify such overlaps, we employed the Graphical Representation of Overlap for Overviews (GROOVE) tool [20], which categorises it into four levels: very high (>15%), high (10–15%), moderate (5–10%), and slight (<5%). If the overlap involves both Cochrane and non-Cochrane reviews, we gave preference to the Cochrane review. For high overlap (≥10%) between two or more non-Cochrane reviews, we gave preference to the MA with the highest AMSTAR 2 score. In the event of identical AMSTAR 2 scores, we gave preference to the MA that included the most RCTs or is the most recent publication. Lastly, for slight or moderate overlap (<10%), we include all MAs (Figures S1−7 in the **Online Supplementary Document**).

### Obtaining data and evaluating its quality

Two reviewers (HT and WM) independently extracted data, with a third reviewer (PT) resolving any disagreements. Specifically, they extracted information on the first author, publication year, sex ratio, age of included participants (year), location, sample size, type of beverage consumption, type of BJD, and main outcome measures. Key outcomes included the incidence of osteoporosis, bone mineral density (BMD) value, serum uric acid (SUA) levels, incidence of gout, incidence of hyperuricemia (HUA), and incidence of RA. The effect measures for our outcomes included relative risks (RRs), odds ratios (ORs), mean difference (MD), standardised MD (SMD), weighted MD, and hazard ratios, along with the effect model (random or fixed) and heterogeneity (*I*^2^ statistic).

Using the AMSTAR 2 tool, two authors (HT and YC) independently assessed the methodological quality of the included MAs, with the senior author (BL) resolving any disagreements [21–23]. We evaluated the evidence level for each outcome using the GRADE system, rating it as high, moderate, low, or very low [24]. Furthermore, we categorised the evidence strength following grading schemes applied in previously published umbrella reviews into five levels: convincing (I), highly suggestive (II), suggestive (III), weak (IV), and non-significant (NS) (**Table 1**) [25,26].

**Table 1:** Evidence classification criteria

Evidence class	Criteria
I	>1000 cases (or >20 000 participants for continuous outcomes); statistical significance at *P* < 10^−6^ (random effects); no evidence of small study effects and excess significance bias; 95% PI excluded null value; no large heterogeneity (*I*^2^ < 50%)
II	>1000 cases (or >20 000 participants for continuous outcomes); statistical significance at *P* < 10^−6^ (random effects); largest study with 95% CI excluding null value
III	>1000 cases (or >20 000 participants for continuous outcomes) and statistical significance at *P* < 0.001
IV	Remaining significant associations with *P* < 0.05
NS	*P* > 0.05

CI – confidence interval, NS – non-significant, PI – prediction interval

### Data analysis

We used systematic reviews and MAs that met the inclusion criteria as the units of analysis and presented their data only. We synthesised the results and displayed the findings from the MAs in tables. After applying the GROOVE tool to assess study overlap, we selected MAs with an overlap rate below 10%. From these MAs, we extracted and pooled quantitative results that met the eligibility criteria for our analysis. We reported dichotomous outcomes as ORs or RRs, and continuous outcomes as MDs or SMDs, all with corresponding 95% confidence intervals (CIs) [27]. We evaluated the degree of heterogeneity among the included studies, not attributable solely to sampling error, using Cochran’s Q and *I*^2^ statistics. We categorised the interpretation of *I*^2^ values as follows: low (*I*^2 ^< 25%), low to moderate (*I*^2^ = 25–50%), moderate to substantial (*I*^2^ = 50–75%), and substantial (*I*^2^>75%) [28]. We performed all meta-MAs using Review Manager, version 5.4 (The Cochrane Collaboration, Copenhagen, Denmark), considering a *P*-value <0.05 statistically significant [29].

## RESULTS

### Search results

Our search retrieved a total of 5900 articles, of which 1968 were duplicates. Based on title and abstract screening using predefined inclusion and exclusion criteria, we excluded 3902 records.

**Figure 1:**
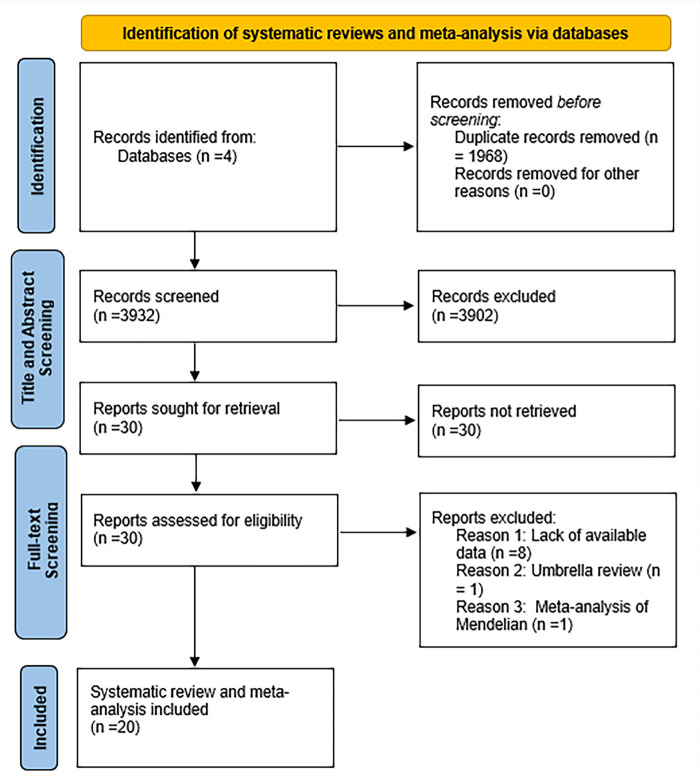
The PRIOR flow diagram of the study selection. PRIOR – Preferred Reporting Items for Overviews of Reviews

We assessed the remaining 30 MAs and systematic reviews in full text, of which we excluded ten, leaving 20 MAs for inclusion in this umbrella review (**Figure 1**).

### Study characteristics

The MAs included in this review were published between 2014 and 2025 (**Table 2**). The average sample size was 525 089. Twelve MAs included >10 studies. All study populations were aged >18 years. More than half (n = 14) of the studies provided specific gender ratios. Nine MAs focussed on the association between beverage consumption and osteoporosis, including tea (n = 6), alcohol (n = 2), and coffee (n = 3); eight examined the relationship between gout and beverage consumption, including SSBs (n = 4), coffee (n = 3), alcohol (n = 1), and tea (n = 1); and four investigated the association between RA and beverage consumption, including tea (n = 3), alcohol (n = 1), and coffee (n = 2). We note here that most of the included studies did not differentiate between tea types; those that did so mainly focused on green tea. Therefore, the results presented here primarily refer to green tea unless otherwise stated.

**Table 2:** Basic information about the studies included

Study, year, and country	Number of studies	% of male participants	% of female participants	Age	Intervention	Condition	Sample size, n	Outcome
Zhang et al., 2017 [10], China	13	NA	NA	NA	Tea	OP	13 078	BMD
Chen et al., 2023 [30], Taiwan	10	NA	NA	25–90*	Coffee	OP	317 412	BMD
Chen et al., 2023 [30], Taiwan	10	NA	NA	34–85*	Tea	OP	247 073	BMD
Guo et al., 2017 [31], China	16	2	98	>19†	Tea	OP	144 047	BMD
Sun et al., 2017 [32], China	17	3.1	96.9	NA	Tea	OP	107 999	IOP
Zhou et al., 2024 [33], China	40	13.5	86.5	≥30†	Tea	OP	893 044	BMD, IOP
Zeng et al., 2022 [34], China	4	11.7	88.3	>20†	Coffee	OP	7114	BMD, IOP
Li et al., 2025 [35], China	14	NA	NA	>40†	Tea + coffee	OP	562 838	IOP
Ke et al., 2023 [15], China	53	NA	NA	≥18†	Alcohol	OP	6 069 770	IOP
Cheraghi et al., 2019 [16], Iran	6	NA	NA	NA	Alcohol	OP	NA	IOP
Zhang et al., 2016 [36], China	11	39	61	>19†	Coffee	Gout	184 751	SUA, IOG, IOH
Park et al., 2016 [37], Korea	9	36.8	63.2	50‡	Coffee	Gout	175 310	SUA, IOG
Li et al., 2018 [38], China	5	35.2	64.8	>30†	Coffee	Gout	156 847	IOG, IOH
Li et al., 2018 [38], China	5	8.2	91.8	≥18†	SSB	Gout	157 974	IOG, IOH
Ayoub-Charette et al., 2021 [39], Canada	26	56.3	43.7	28.9 (9.1)‡	SSB	Gout	867	SUA
Ayoub-Charette et al., 2019 [12], Canada	2	37	63	50.2‡	SSB	Gout	125 299	IOG
Ebrahimpour-Koujan et al., 2020 [13], Iran	8	36.7	63.3	17–75*	SSB	Gout	194 019	IOG, IOH
Zhang et al., 2017 [40], China	15	39.7	60.3	50.3‡	Tea	Gout	197 465	SUA, IOG, IOH
Asoudeh et al., 2022 [41], Iran	5	4.6	95.4	20–98*	Tea + coffee	RA	266 985	IORA
Lee et al., 2014 [42], Korea	5	7.5	92.5	50.6‡	Tea + coffee	RA	134 989	IORA
Long et al., 2023 [43], China	2	NA	NA	50.8‡	Tea	RA	164	DAS28, CRP
Wieczorek et al., 2022 [44], UK	14	59.2	17.8	69.3‡	Alcohol	RA + gout	19 664	SUA, DAS28

BMD – bone mineral density, CRP – C-reactive protein, DAS28 – Disease Activity Score 28, IOA – the incidence of osteoarthritis, IOG – the incidence of gout, IOH – the incidence of hyperuricemia, IOP – the incidence of osteoporosis, IORA – the incidence of RA, NA – no access, OA – osteoarthritis, OP – osteoporosis, SUA – serum uric acid

*Reported as range.

†Reported as threshold.

‡Reported as mean or mean (standard deviation).

We evaluated the quality of each MA using AMSTAR 2 and assessed the certainty of evidence using GRADE (Tables S3−9 in the **Online Supplementary Document**). Per the GRADE assessment, the quality of evidence across most outcomes was rated as low to very low, primarily due to serious risk of bias, imprecision, and publication bias. For osteoporosis and BMD, most studies on tea, coffee, and alcohol showed very low certainty, mainly due to methodological limitations and suspected publication bias. For SUA, studies on SSBs provided high to moderate certainty, while those on coffee and tea were of low to very low certainty. Evidence on the incidence of gout and hyperuricemia was mostly of low or very low certainty, especially for coffee and tea, with SSBs showing slightly higher quality in some cases. For rheumatoid arthritis, coffee-related studies were rated as low certainty, whereas tea-related evidence was mostly very low, due to additional concerns such as indirectness.

### Osteoporosis outcomes

#### Incidence of osteoporosis

**Figure 2:**
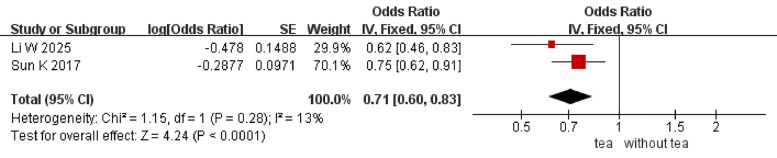
Meta-meta-analysis of the association between tea consumption and osteoporosis incidence.

Four MAs explored the association between osteoporosis incidence and the consumption of tea and coffee (Table S10 in the **Online Supplementary Document**) [32–35]. For tea, three meta-analyses were included, with two reporting dichotomous outcomes using ORs and one using RRs. Because the GROOVE tool evaluates study overlap based on consistent effect measures, we applied it only to the two meta-analyses that reported ORs. As our analysis provides a higher level of evidence synthesis, we reported only the results derived from this analysis and did not separately report the findings from the meta-analysis that used RRs. The pooled analysis based on a fixed-effects model demonstrated that tea consumption significantly reduced the incidence of osteoporosis (OR = 0.71; 95% CI = 0.60, 0.83; *P* < 0.0001; *I*^2^ = 13%) (**Figure 2**). Based on the GROOVE analysis, we retained the one with the most representative and least overlapping evidence [35] and excluded the other due to substantial overlap [34]. The authors found that consuming coffee does not significantly lower the incidence of osteoporosis (OR = 0.79; 95% CI = 0.73, 0.84; *P* = 0.17; *I*^2^ = 28.9%; low level of evidence; evidence strength = IV). One MA also examined the relationship between alcohol consumption and the incidence of osteoporosis. They found that alcoholic beverages could elevate the risk of osteoporosis (OR = 2.95; 95% CI = 1.78, 4.9; *P* < 0.0001; *I*^2^ = 0%; low level of evidence; evidence strength = III) [16].

#### BMD values

Four MAs with six results explored the association between BMD values and tea consumption (Table S12 in the **Online Supplementary Document**) [10,30,31,33]. We used the GROOVE tool to identify overlap among these three MAs, using MD as the statistical metric. Based on the strategies for resolving overlap described in the methods, we considered the result from one study to provide the most complete overview of the available evidence [30]. Its authors found no statistically significant difference in BMD values between tea drinkers and non-tea drinkers (MD = 0.039; 95% CI = 0.012, 0.09; *P* = 0.132; *I*^2^ = 98.455%; very low levels of evidence; evidence strength = NS). However, one MA found that drinking tea significantly increased BMD values (OR = 0.66; 95% CI = 0.47, 0.94; *P* = 0.02; *I*^2^ = 46%; low level of evidence; evidence strength = IV) [10]. Similarly, another MA reported a significant increase in BMD values (SMD = 0.332; 95% CI = 0.207, 0.457; *P* < 0.05; *I*^2^ = 94%; very low level of evidence; evidence strength = IV) [33]. One MA explored the association between BMD values and coffee consumption. It found no statistically significant difference in BMD values between coffee drinkers and non-coffee drinkers (MD = 0.02; 95% CI = −0.003, 0.044; *P* = 0.093; *I*^2^ = 0%; very low level of evidence; evidence strength = NS) [30].

### Gout outcomes

#### SUA values

Four MAs with six results have explored the association between beverage consumption and SUA levels (Table S3 in the **Online Supplementary Document**) [36,37,39,40]. In the MA that examined SSBs, two results demonstrated a positive correlation between SSB consumption and elevated SUA levels — one from the substitution design (MD = 0.42; 95% CI = 0.24, 0.59; *P* < 0.01; *I*² = 33%; high level of evidence; evidence strength = IV) and one from the addition design (MD = 0.43; 95% CI = 0.23, 0.63; *P* < 0.01; *I*² = 56.17%; high level of evidence; evidence strength = IV). In contrast, one MA result based on the subtraction design demonstrated no significant association (MD = 0.09; 95% CI = −0.14, 0.32; *P* = 0.45; *I*² = 0%; moderate level of evidence; evidence strength = NS) [39]. In two MAs that examined coffee consumption, one MA found that drinking coffee was not statistically significantly associated with an increase in SUA levels (SMD = −0.09; 95% CI = −0.23, 0.05; *P* = 0.21; *I*^2^ = 84.2%; low level of evidence; evidence strength = NS) [40]. The other one reported a similar result (MD = −0.09; 95% CI = −0.43, 0.25; *P* = 0.61; *I*^2^ = 97%; evidence level = very low; evidence strength = NS) [37]. One MA reported no statistically significant association between tea consumption and SUA levels (weighted MD = 7.41; 95% CI = −2.34, 17.15; *P* = 0.136; *I*^2^ = 92.5%; very low level of evidence; evidence strength = NS) [36].

#### The incidence of gout

Four MAs with six results explored the association between beverage consumption and the incidence of gout (Table S13 in the **Online Supplementary Document**) [12,13,38,40]. We conducted a GROOVE analysis for SSB consumption, using RRs as the statistical metric. Based on the strategies for resolving overlap issues, we considered one MA to encompass the most available evidence. They indicated that SSB consumption is statistically associated with an increased incidence of gout, with separate pooled results reported for different study designs: cohort studies (RR = 1.35; 95% CI = 1.18, 1.55; *P* < 0.05; *I*² = 40.1%; very low level of evidence; evidence strength = IV) and case-control and cross-sectional studies (RR = 1.33; 95% CI = 1.06, 1.66; *P* < 0.05; *I*² = 0%; very low level of evidence; evidence strength = IV) [13]. The other MA that used ORs as the main metric reached the same conclusion (OR = 2.14; 95% CI = 1.65, 2.78; *P* < 0.01; *I*^2^ = 0%; low level of evidence; evidence strength = I) [38]. For coffee consumption, one MA found that coffee consumption was associated with a lower risk of incident gout (RR = 0.43; 95% CI = 0.31, 0.59; *P* < 0.001; *I*^2^ = 0%; very low level of evidence; evidence strength = III) [40]. Another MA arrived at same conclusions (OR = 0.47; 95% CI = 0.37, 0.59; *P* < 0.01; *I*^2^ = 39%; very low level of evidence; evidence strength = I) [38]. However, no specific relation was found between alcoholic beverages and BJDs.

#### The incidence of HUA

Four MAs with five results explored the association between beverage consumption and the incidence of HUA (Table S14 in the **Online Supplementary Document**) [13,36,38,40]. One MA indicated that SSB consumption is statistically associated with an increased incidence of HUA (RR = 1.35; 95% CI = 1.19, 1.52; *P* < 0.05; *I*^2^ = 41.4%; low level of evidence; evidence strength = IV) [13]. Another study reached the same conclusion (OR = 1.85; 95% CI = 1.66, 2.07; *P* < 0.01; *I*^2^ = 31%; moderate level of evidence; evidence strength = I) [38]. For coffee consumption, we conducted a GROOVE analysis using RR as the statistical metric. Based on the strategies for resolving overlap issues, we considered one MA to encompass most of the available evidence. The authors reported that drinking coffee is not statistically significantly associated with a decrease in the incidence of HUA (OR = 0.96; 95% CI = 0.76, 1.22; *P* = 0.75; *I*^2^ = 52%; very low level of evidence; evidence strength = NS) [38]. One MA mentioned the association between tea consumption and the incidence of HUA, but found no significant association (OR = 0.98; 95% CI = 0.77, 1.24; *P* = 0.839; *I*^2^ = 72.3%; very low level of evidence; evidence strength = NS) [36].

### RA outcome

Two MAs assessed the association between beverage consumption and the incidence of RA (Table S15 in the **Online Supplementary Document**) [41,42]. Both focussed on outcomes related to coffee and tea consumption. Based on the strategies for resolving overlap issues, we considered one MA to be most comprehensive in terms of encompassed evidence [41]. The authors indicated that coffee consumption might significantly increase the incidence of RA (RR = 1.3; 95% CI = 1.04, 1.62; *P* < 0.05, *I*^2^ = 0%; low level of evidence; evidence strength = IV), but found no significant association between tea consumption and the incidence of RA (RR = 1.05; 95% CI = 0.73, 1.53; *P* > 0.05; *I*^2^ = 69.1%; low level of evidence; evidence strength = NS). We note here that the original MA did not provide an exact *P*-value, nor could it be derived due to the lack of access to raw data. No studies presented data on the effects of alcohol and SSBs on RA.

## DISCUSSION

In this umbrella review, we found heterogeneous associations between various types of beverage consumption and BJDs. Tea consumption was consistently associated with a reduced risk of osteoporosis, suggesting that it may have protective effects on bone health. In contrast, alcohol intake was positively associated with osteoporosis risk, while SSBs were linked to an increased risk of gout. We observed no significant associations between coffee intake and major BJDs. These findings highlight the need to consider the type of beverage when evaluating dietary risk factors for BJDs.

### Tea

Tea consumption appears to have a beneficial effect on osteoporosis-related conditions. Tea is a beverage rich in various bioactive components, with its polyphenols being the primary contributor to its health benefits. Mechanistically, these polyphenols have been shown to modulate uric acid metabolism in mice by inhibiting xanthine oxidase, the enzyme responsible for uric acid production, suggesting a potential role in reducing HUA and mitigating gout symptoms [45]. Among them, epigallocatechin gallate is particularly abundant and has been reported to enhance alkaline phosphatase activity, increase protein and gene expression in osteoblast-like cells, induce apoptosis in osteoclasts, and promote mineralised bone formation [46,47]. Moreover, epigallocatechin gallate inhibits the activation of nuclear factor-κB, a key signalling pathway involved in inflammation and bone resorption, which may contribute to reduced inflammation-driven bone loss in conditions such as RA [48]. Tea polyphenols have also been shown to promote osteoblast survival, possibly by increasing circulating vitamin D levels, reducing osteoblast apoptosis, and enhancing cell proliferation and differentiation [49–52]. One study reported that catechin treatment increased alkaline phosphatase activity and reduced tumour necrosis factor-α-induced apoptosis in osteoblasts [49]. In addition, tea extracts have been shown to protect mononuclear cells from oxidative stress, which is considered to contribute to bone degradation [53,54]. Tea also contains minerals such as calcium and phosphorus, as well as vitamins C and K, which have been implicated in supporting bone health. Although some studies have raised concerns that caffeine in tea may negatively affect bone metabolism, the caffeine content in most teas is relatively low (2–4%) [32]. Consequently, the risk of BJD due to caffeine in tea is relatively low [55]. One study concluded that consuming 1–1.5 cups of tea per day provides the most significant protective effect against osteoporosis [33]. However, consumption of strong tea has been associated with a higher incidence of osteoporosis in some studies, potentially due to the high caffeine content [32]. Thus, the impact of tea dosage and type warrants further investigation. Some evidence suggests a potential benefit of tea in managing gout, particularly green tea, which was found to be significantly associated with symptom improvement in a study [36]. Additionally, although several studies, including RCTs, have indicated that tea may have a positive effect on RA, these findings are limited by small sample sizes and require further experimental validation [41-43]. Therefore, due to factors such as the variety of tea, dosage, and limited sample size, the relationship between tea and BJDs beyond osteoporosis, such as gout and RA, requires more rigorous investigation.

### Coffee

We found no significant association between coffee consumption and BJDs. This relationship remains inconsistent across studies, potentially due to the complex biochemical properties of its diverse bioactive components. Among these, caffeine has been extensively studied for its possible adverse effects on bone and muscle health. One study demonstrated that moderate to high concentrations of caffeine (1–10 mM) can enhance osteoclast differentiation in a dose-dependent manner, possibly by regulating intracellular calcium levels during osteoclast genesis or by reducing vitamin D receptor expression, thereby affecting bone metabolism [55]. Caffeine may also influence inflammatory responses. Some reported that long-term consumption of high levels of coffee is associated with elevated circulating interleukin-6, an inflammatory biomarker [56]. Additionally, caffeine has been shown to increase adiponectin levels, which may promote inflammation by stimulating the release of pro-inflammatory mediators [57]. However, caffeine is not the only biologically active component in coffee. Other compounds, such as tannins, may exert protective effects. Animal studies have shown that tannins in coffee can support skeletal health in rats [30]. Moreover, coffee is rich in polyphenols, similar to those found in tea, which may help enhance bone density by mitigating oxidative stress and suppressing bone resorption. For example, one study reported that chlorogenic acid, a polyphenol present in coffee, inhibits xanthine oxidase activity, enhances renal blood flow, and promotes uric acid excretion, thus potentially reducing the risk of developing gout [58]. The inconsistencies across studies investigating coffee and BJDs may also be attributed to differences in coffee consumption levels. Some evidence suggests that moderate coffee intake may strike a balance between beneficial and harmful components. Furthermore, heterogeneity in factors such as participant age, sex, coffee type (*e.g.* sugar-sweetened or milk-added), regional dietary habits, and lifestyle factors like alcohol use and smoking may further contribute to the varied findings [34,37,41].

### SSBs

We found that SSBs have a detrimental impact on BJDs, particularly gout, which is consistent with previous literature [12,13,39]. The primary contributor to this effect is the added fructose. Excessive fructose intake can elevate SUA levels through the unregulated fructose kinase pathway, in which large amounts of ATP are consumed to convert fructose into fructose-1-phosphate in the liver [59]. The resulting ATP depletion leads to AMP accumulation, which is subsequently metabolised into uric acid. Moreover, fructose can enhance *de novo* purine synthesis, further contributing to HUA [60]. These mechanisms clearly explain how excessive fructose intake can impair uric acid metabolism, highlighting the importance of reducing SSB consumption for gout prevention and management. In addition to gout, several studies have suggested that high intake of SSBs may also adversely affect skeletal health. An umbrella review summarising the effects of dietary sugar intake reported that SSBs are associated with reduced bone quality and altered bone metabolism [14]. This may be attributed to the impact of fructose on calcium homeostasis. Specifically, high fructose consumption has been linked to decreased intestinal calcium absorption, potentially through alterations in gut microbiota composition or the downregulation of calcium transporters. Furthermore, phosphoric acid – commonly found in many soft drinks – may disrupt calcium-phosphate balance and exacerbate calcium loss [61]. The low pH of carbonated beverages such as cola may also impair calcium absorption by affecting gastric acidity [62]. These factors may contribute to impaired bone metabolism. Currently, there is limited evidence regarding the association between SSB consumption and other BJDs such as RA, so this topic warrants further research.

### Alcohol

The most recent MA reported a J-shaped nonlinear association between alcohol consumption and bone health, with alcohol intake up to 22 g per day showing potential benefits, while intake above 40 g per day significantly increased the risk of adverse skeletal outcomes [15]. This finding aligns that of other studies [63]. The protective effects at low levels of consumption may be attributed to alcohol-induced stimulation of calcitonin secretion, which inhibits bone resorption and contributes to improved BMD [64]. However, chronic excessive alcohol intake has detrimental effects on bone metabolism. It impairs osteoblast function by promoting oxidative stress and inhibiting the Wnt signalling pathway, ultimately leading to bone loss [65]. Excessive alcohol intake can also stimulate adipogenesis, suppress osteogenic activity in the bone marrow stroma, and induce osteocyte apoptosis [66]. Moreover, alcohol disrupts the absorption of calcium and vitamin D and alters the hormonal environment, particularly reducing levels of sex hormones, which collectively contribute to decreased BMD [67,68]. Beyond its effects on bone, alcohol consumption is recognised as a risk factor for joint-related conditions such as RA and gout. Alcohol can exacerbate systemic inflammation by promoting the release of pro-inflammatory cytokines, thereby aggravating joint damage. It may also interfere with uric acid metabolism, increasing SUA levels and triggering gout attacks [44]. Furthermore, long-term alcohol use is associated with increased adiposity and body weight, which can elevate mechanical stress on joints and contribute to the development or progression of arthritis. Obesity related to chronic alcohol intake has also been shown to negatively impact joint function and recovery, particularly following joint replacement surgeries. Overall, alcohol consumption plays a significant role in the pathogenesis and progression of various BJDs, especially when consumed in excess.

### Study limitations

This is the first umbrella review to summarise evidence from existing MAs on the association between beverage consumption and BJDs. However, several limitations should be acknowledged. First, the number of original MAs included in this umbrella review was insufficient. In detail, umbrella reviews rely on existing MAs, but for some specific beverage-disease pairs, only one or two such MAs exist. This limits the strength and reliability of the conclusions for those combinations. Further research is needed on the relationship between beverage consumption and bone and joint diseases, as well as its potential impact on postoperative recovery. A second limitation is the lack of RCTs in the included Mas, as they mainly consisted of cross-sectional and prospective cohort studies, highlighting a need for experimental studies that would help establish causality. Another significant concern is the lack of data for performing a subgroup analysis. Various original studies have noted that potential confounding factors, such as gender differences, varying beverage dosages, follow-up durations, causes of osteoporotic fracture, geographic differences, and the proportion of different chemicals in the beverages, which might have introduced bias into the results. However, after categorising the MAs by disease and beverage type, we were unable to conduct subgroup analyses to account for these potential confounders. Lastly, several studies included in the analysis reported surrogate outcomes such as Disease Activity Score-28 and C-reactive protein. While these measures are commonly used to reflect disease activity, particularly in conditions such as RAs, they do not directly represent clinical endpoints such as disease incidence, progression, or quality of life. Consequently, any findings based on these surrogate outcomes may be of limited use in clinical contexts. Further research is needed to clarify the associations between beverage consumption and clinically relevant outcomes beyond surrogate biomarkers.

## CONCLUSIONS

Beverage consumption shows varying associations with BJDs. Tea may protect against osteoporosis, while alcohol and SSBs could be linked to adverse outcomes. No clear association was found for coffee. These findings support the need for bone and joint health-oriented dietary guidance.

## Additional material


Online Supplementary Document
