# Supplementary material for: Beverage consumption on bone and joint disorders: an umbrella review
Source: J Glob Health. 2025 Aug 4;15:04222. doi: 10.7189/jogh.15.04222 (PMC12319397; doi:10.7189/jogh.15.04222)
Supplement: Online Supplementary Document [file jogh-15-04222-s001.pdf]

Supplement to: Tan H, Tang P, Chai H, Ma W, Cao Y, Lin B, Zhu Y, Xiao W, Wen T, Lu B, Li Y. Beverage consumption on bone and joint disorders: an umbrella review. J Glob Health. 2025;15:04222.

Table S1: PRIOR Checklist

(Gates M, Gates A, Pieper D, et al. Reporting guideline for overviews of reviews of healthcare interventions: development of the PRIOR statement. *BMJ* 2022;378:e070849. doi:10.1136/bmj-2022-070849.)

**Section** Topic	#	**Item**	**Location reported**
**TITLE**			
Title	1	Identify the report as an overview of reviews.	Page 1
**ABSTRACT**			
Abstract	2	Provide a comprehensive and accurate summary of the purpose, methods, and results of the overview of reviews.	Page 1-2
**INTRODUCTION**			
Rationale	3	Describe the rationale for conducting the overview of reviews in the context of existing knowledge.	Page 2-3
Objectives	4	Provide an explicit statement of the objective(s) or question(s) addressed by the overview of reviews.	Page 3
**METHODS**			
Eligibility criteria	5a	Specify the inclusion and exclusion criteria for the overview of reviews. If supplemental primary studies were included, this should be stated, with a rationale.	Page 4
	5b	Specify the definition of 'systematic review' as used in the inclusion criteria for the overview of reviews.	Page 4
Information sources	6	Specify all databases, registers, websites, organizations, reference lists, and other sources searched or consulted to identify systematic reviews and supplemental primary studies (if included). Specify the date when each source was last searched or consulted.	Page 3
Search strategy	7	Present the full search strategies for all databases, registers and websites, such that they could be reproduced. Describe any search filters and limits applied.	Page 3
Selection process	8a	Describe the methods used to decide whether a systematic review or supplemental primary study (if included) met the inclusion criteria of the overview of reviews.	Page 4
	8b	Describe how overlap in the populations, interventions, comparators, and/or outcomes of systematic reviews was identified and managed during study selection.	Page 4-5
Data collection process	9a	Describe the methods used to collect data from reports.	Page 5
	9b	If applicable, describe the methods used to identify and manage primary study overlap at the level of the comparison and outcome during data collection. For each outcome, specify the method used to illustrate and/or quantify the degree of primary study overlap across systematic reviews.	Page 4-5
	9c	If applicable, specify the methods used to manage discrepant data across systematic reviews during data collection.	Page 4-5
Data items	10	List and define all variables and outcomes for which data were sought. Describe any assumptions made and/or measures taken to identify and clarify missing or unclear information.	Page 5
Risk of bias assessment	11a	Describe the methods used to *assess* risk of bias or methodological quality of the included systematic reviews.	Page 5
	11b	Describe the methods used to *collect* data on (from the systematic reviews) and/or *assess* the risk of bias of the primary studies included in the systematic reviews. Provide a justification for instances where flawed, incomplete, or missing assessments are identified but not re-assessed.	Page 5
	11c	Describe the methods used to *assess* the risk of bias of supplemental primary studies (if included).	Page 5
Synthesis methods	12a	Describe the methods used to summarize or synthesize results and provide a rationale for the choice(s).	Page 6
	12b	Describe any methods used to explore possible causes of heterogeneity among results.	Page 6
	12c	Describe any sensitivity analyses conducted to assess the robustness of the synthesized results.	Page 6

Reporting bias assessment	13	Describe the methods used to *collect* data on (from the systematic reviews) and/or *assess* the risk of bias due to missing results in a summary or synthesis (arising from reporting biases at the levels of the systematic reviews, primary studies, and supplemental primary studies, if included).	Page 6
Certainty assessment	14	Describe the methods used to *collect* data on (from the systematic reviews) and/or *assess* certainty (or confidence) in the body of evidence for an outcome.	Page 6
**RESULTS**			
Systematic review and supplemental primary study selection	15a	Describe the results of the search and selection process, including the number of records screened, assessed for eligibility, and included in the overview of reviews, ideally with a flow diagram.	Page 6-7
	15b	Provide a list of studies that might appear to meet the inclusion criteria, but were excluded, with the main reason for exclusion.	Page 6

**Section** Topic	#	**Item**	**Location reported**
Characteristics of systematic reviews and supplemental primary studies	16	Cite each included systematic review and supplemental primary study (if included) and present its characteristics.	Page 7-8
Primary study overlap	17	Describe the extent of primary study overlap across the included systematic reviews.	Page 9-13
Risk of bias in systematic reviews, primary studies, and supplemental primary studies	18a	Present assessments of risk of bias or methodological quality for each included systematic review.	Page 8
	18b	Present assessments (*collected* from systematic reviews or *assessed* anew) of the risk of bias of the primary studies included in the systematic reviews.	Page 8
	18c	Present assessments of the risk of bias of supplemental primary studies (if included).	Page 8
Summary or synthesis of results	19a	For all outcomes, summarize the evidence from the systematic reviews and supplemental primary studies (if included). If meta-analyses were done, present for each the summary estimate and its precision and measures of statistical heterogeneity. If comparing groups, describe the direction of the effect.	Page 9-13
	19b	If meta-analyses were done, present results of all investigations of possible causes of heterogeneity.	Page 9
	19c	If meta-analyses were done, present results of all sensitivity analyses conducted to assess the robustness of synthesized results.	Page 9
Reporting biases	20	Present assessments (*collected* from systematic reviews and/or *assessed* anew) of the risk of bias due to missing primary studies, analyses, or results in a summary or synthesis (arising from reporting biases at the levels of the systematic reviews, primary studies, and supplemental primary studies, if included) for each summary or synthesis assessed.	Page 9-13
Certainty of evidence	21	Present assessments (*collected* or *assessed* anew) of certainty (or confidence) in the body of evidence for each outcome.	Page 9-13
**DISCUSSION**			
Discussion	22a	Summarize the main findings, including any discrepancies in findings across the included systematic reviews and supplemental primary studies (if included).	Page 13
	22b	Provide a general interpretation of the results in the context of other evidence.	Page 14-18
	22c	Discuss any limitations of the evidence from systematic reviews, their primary studies, and supplemental primary studies (if included) included in the overview of reviews. Discuss any limitations of the overview of reviews methods used.	Page 18-19
	22d	Discuss implications for practice, policy, and future research (both systematic reviews and primary research). Consider the relevance of the findings to the end users of the overview of reviews, e.g., healthcare providers, policymakers, patients, among others.	Page 14-19

OTHER INFORMATION			
Registration and protocol	23a	Provide registration information for the overview of reviews, including register name and registration number, or state that the overview of reviews was not registered.	Page 3
	23b	Indicate where the overview of reviews protocol can be accessed, or state that a protocol was not prepared.	Page 3
	23c	Describe and explain any amendments to information provided at registration or in the protocol. Indicate the stage of the overview of reviews at which amendments were made.	No
Support	24	Describe sources of financial or non-financial support for the overview of reviews, and the role of the funders or sponsors in the overview of reviews.	Page 21-22
Competing interests	25	Declare any competing interests of the overview of reviews' authors.	Page 21
Author information	26a	Provide contact information for the corresponding author.	Page 22
	26b	Describe the contributions of individual authors and identify the guarantor of the overview of reviews.	Page 21
Availability of data and other materials	27	Report which of the following are available, where they can be found, and under which conditions they may be accessed: template data collection forms; data collected from included systematic reviews and supplemental primary studies; analytic code; any other materials used in the overview of reviews.	Page 21

Text S1: the literature search query

Pubmed

Gout and tea

((((((((((tea[MeSH Terms]) OR (tea[Title/Abstract])) OR (Black Tea[Title/Abstract])) OR (Black Teas[Title/Abstract])) OR (Tea, Black[Title/Abstract])) OR (Teas, Black[Title/Abstract])) OR (Green Tea[Title/Abstract])) OR (Green Teas[Title/Abstract])) OR (Tea, Green[Title/Abstract])) OR (Teas, Green[Title/Abstract])) AND (((gout[Title/Abstract]) OR (gout[MeSH Terms])) OR (gouts[Title/Abstract])) ——————46

Osteoarthritis and tea

((((((((((((Osteoarthritis[Title/Abstract]) OR (Osteoarthritis[MeSH Terms])) OR (Osteoarthritides[Title/Abstract])) OR (Osteoarthrosis[Title/Abstract])) OR (Osteoarthroses[Title/Abstract])) OR (Arthritis, Degenerative[Title/Abstract])) OR (Arthritides, Degenerative[Title/Abstract])) OR (Degenerative Arthritides[Title/Abstract])) OR (Degenerative Arthritis[Title/Abstract])) OR (Arthrosis[Title/Abstract])) OR (Arthroses[Title/Abstract])) OR (Osteoarthrosis Deformans[Title/Abstract])) AND ((((((((((tea[MeSH Terms]) OR (tea[Title/Abstract])) OR (Black Tea[Title/Abstract])) OR (Black Teas[Title/Abstract])) OR (Tea, Black[Title/Abstract])) OR (Teas, Black[Title/Abstract])) OR (Green Tea[Title/Abstract])) OR (Green Teas[Title/Abstract])) OR (Tea, Green[Title/Abstract])) OR (Teas, Green[Title/Abstract])) ——————176

Rheumatoid Arthritis and tea

((Rheumatoid Arthritis[Title/Abstract]) OR (Rheumatoid Arthritis[MeSH Terms])) AND ((((((((((tea[MeSH Terms]) OR (tea[Title/Abstract])) OR (Black Tea[Title/Abstract])) OR (Black Teas[Title/Abstract])) OR (Tea, Black[Title/Abstract])) OR (Teas, Black[Title/Abstract])) OR (Green Tea[Title/Abstract])) OR (Green Teas[Title/Abstract])) OR (Tea, Green[Title/Abstract])) OR (Teas, Green[Title/Abstract])) ——————184

Osteoporosis and tea

((((((((((((((((((((((Osteoporosis[MeSH Terms]) OR (Osteoporosis[Title/Abstract])) OR (Osteoporoses[Title/Abstract])) OR (Osteoporosis, Post-Traumatic[Title/Abstract])) OR (Osteoporosis, Post Traumatic[Title/Abstract])) OR (Post-Traumatic Osteoporoses[Title/Abstract])) OR (Post-Traumatic Osteoporosis[Title/Abstract])) OR (Osteoporosis, Senile[Title/Abstract])) OR (Osteoporoses, Senile[Title/Abstract])) OR (Senile Osteoporoses[Title/Abstract])) OR (Osteoporosis, Involutional[Title/Abstract])) OR (Senile Osteoporosis[Title/Abstract])) OR (Osteoporosis, Age-Related[Title/Abstract])) OR (Osteoporosis, Age Related[Title/Abstract])) OR (Bone Loss, Age-Related[Title/Abstract])) OR (Age-Related Bone Loss[Title/Abstract])) OR (Age-Related Bone Losses[Title/Abstract])) OR (Bone Loss, Age Related[Title/Abstract])) OR (Bone Losses, Age-Related[Title/Abstract])) OR (Age-Related Osteoporosis[Title/Abstract])) OR (Age Related Osteoporosis[Title/Abstract])) OR (Age-Related Osteoporoses[Title/Abstract])) OR (Osteoporoses, Age-Related[Title/Abstract])) AND ((((((((((tea[MeSH Terms]) OR (tea[Title/Abstract])) OR (Black Tea[Title/Abstract])) OR (Black Teas[Title/Abstract])) OR (Tea, Black[Title/Abstract])) OR (Teas, Black[Title/Abstract])) OR (Green Tea[Title/Abstract])) OR (Green Teas[Title/Abstract])) OR (Tea, Green[Title/Abstract])) OR (Teas, Green[Title/Abstract])) ——————211

Gout and coffee

(((gout[Title/Abstract]) OR (gout[MeSH Terms])) OR (gouts[Title/Abstract])) AND ((coffee[Title/Abstract]) OR (coffee[MeSH Terms]))——————55

Osteoarthritis and coffee

((((((((((((Osteoarthritis[Title/Abstract]) OR (Osteoarthritis[MeSH Terms])) OR (Osteoarthritides[Title/Abstract])) OR (Osteoarthrosis[Title/Abstract])) OR (Osteoarthroses[Title/Abstract])) OR (Arthritis, Degenerative[Title/Abstract])) OR (Arthritides, Degenerative[Title/Abstract])) OR (Degenerative Arthritides[Title/Abstract])) OR (Degenerative Arthritis[Title/Abstract])) OR (Arthrosis[Title/Abstract])) OR (Arthroses[Title/Abstract])) OR (Osteoarthrosis Deformans[Title/Abstract])) AND ((coffee[Title/Abstract]) OR (coffee[MeSH Terms]))——————26

Rheumatoid Arthritis and coffee

((Rheumatoid Arthritis[Title/Abstract]) OR (Rheumatoid Arthritis[MeSH Terms])) AND ((coffee[Title/Abstract]) OR (coffee[MeSH Terms]))——————57

Osteoporosis and coffee

(((((((((((((((((((((((Osteoporosis[MeSH Terms]) OR (Osteoporosis[Title/Abstract])) OR (Osteoporoses[Title/Abstract])) OR (Osteoporosis, Post-Traumatic[Title/Abstract])) OR (Osteoporosis, Post Traumatic[Title/Abstract])) OR (Post-Traumatic Osteoporoses[Title/Abstract])) OR (Post-Traumatic Osteoporosis[Title/Abstract])) OR (Osteoporosis, Senile[Title/Abstract])) OR (Osteoporoses, Senile[Title/Abstract])) OR (Senile Osteoporoses[Title/Abstract])) OR (Osteoporosis, Involutional[Title/Abstract])) OR (Senile Osteoporosis[Title/Abstract])) OR (Osteoporosis, Age-Related[Title/Abstract])) OR (Osteoporosis, Age Related[Title/Abstract])) OR (Bone Loss, Age-Related[Title/Abstract])) OR (Age-Related Bone Loss[Title/Abstract])) OR (Age-Related Bone Losses[Title/Abstract])) OR (Bone Loss, Age Related[Title/Abstract])) OR (Bone Losses, Age-Related[Title/Abstract])) OR (Age-Related Osteoporosis[Title/Abstract])) OR (Age Related Osteoporosis[Title/Abstract])) OR (Age-Related Osteoporoses[Title/Abstract])) OR (Osteoporoses, Age-Related[Title/Abstract])) AND ((coffee[Title/Abstract]) OR (coffee[MeSH Terms])) ———152

Gout and SSBs

(((gout[Title/Abstract]) OR (gout[MeSH Terms])) OR (gouts[Title/Abstract])) AND (((((((((((((((((((((((((((((((((((((((Sugar-Sweetened Beverages[MeSH Terms]) OR (Sugar-Sweetened Beverages[Title/Abstract])) OR (Beverage, Sugar-Sweetened[Title/Abstract])) OR (Beverages, Sugar-Sweetened[Title/Abstract])) OR (Sugar-Sweetened Beverage[Title/Abstract])) OR (Sugar-Added Beverages[Title/Abstract])) OR (Sugar Added Beverages[Title/Abstract])) OR (Sugar Sweetened Beverage[Title/Abstract])) OR (Beverage, Sugar Sweetened[Title/Abstract])) OR (Beverages, Sugar Sweetened[Title/Abstract])) OR (Sugar Sweetened Beverages[Title/Abstract])) OR (Sweetened Beverage, Sugar[Title/Abstract])) OR (Sweetened Beverages, Sugar[Title/Abstract])) OR (Sugar-Added Beverage[Title/Abstract])) OR (Beverage, Sugar-Added[Title/Abstract])) OR (Beverages, Sugar-Added[Title/Abstract])) OR (Sugar Added Beverage[Title/Abstract])) OR (Sweetened Drinks[Title/Abstract])) OR (Sweetened Drink[Title/Abstract])) OR (Drink, Sweetened[Title/Abstract])) OR (Drinks, Sweetened[Title/Abstract])) OR (Sugar-Sweetened Soft Drinks[Title/Abstract])) OR (Sugar Sweetened Soft Drinks[Title/Abstract])) OR (Sugar-Sweetened Soft Drink[Title/Abstract])) OR (Drink, Sugar-Sweetened Soft[Title/Abstract])) OR (Drinks, Sugar-Sweetened Soft[Title/Abstract])) OR (Soft Drink, Sugar-Sweetened[Title/Abstract])) OR (Soft Drinks, Sugar-Sweetened[Title/Abstract])) OR (Sugar Sweetened Soft Drink[Title/Abstract])) OR (Sweetened Beverages[Title/Abstract])) OR (Sweetened Beverage[Title/Abstract])) OR (Beverage, Sweetened[Title/Abstract])) OR (Beverages, Sweetened[Title/Abstract])) OR (Sugar-Sweetened Sodas[Title/Abstract])) OR (Sugar Sweetened Sodas[Title/Abstract])) OR (Sugar-Sweetened Soda[Title/Abstract])) OR (Soda, Sugar-Sweetened[Title/Abstract])) OR (Sodas, Sugar-Sweetened[Title/Abstract])) OR (Sugar Sweetened Soda[Title/Abstract]))———
———39

Osteoarthritis and SSBs

((((((((((((Osteoarthritis[Title/Abstract]) OR (Osteoarthritis[MeSH Terms])) OR (Osteoarthritides[Title/Abstract])) OR (Osteoarthrosis[Title/Abstract])) OR (Osteoarthroses[Title/Abstract])) OR (Arthritis, Degenerative[Title/Abstract])) OR (Arthritides, Degenerative[Title/Abstract])) OR (Degenerative Arthritides[Title/Abstract])) OR (Degenerative Arthritis[Title/Abstract])) OR (Arthrosis[Title/Abstract])) OR (Arthroses[Title/Abstract])) OR (Osteoarthrosis Deformans[Title/Abstract])) AND (((((((((((((((((((((((((((((((((((((((Sugar-Sweetened Beverages[MeSH Terms]) OR (Sugar-Sweetened Beverages[Title/Abstract])) OR (Beverage, Sugar-Sweetened[Title/Abstract])) OR (Beverages, Sugar-Sweetened[Title/Abstract])) OR (Sugar-Sweetened Beverage[Title/Abstract])) OR (Sugar-Added Beverages[Title/Abstract])) OR (Sugar Added Beverages[Title/Abstract])) OR (Sugar Sweetened Beverage[Title/Abstract])) OR (Beverage, Sugar Sweetened[Title/Abstract])) OR (Beverages, Sugar Sweetened[Title/Abstract])) OR (Sugar Sweetened Beverages[Title/Abstract])) OR (Sweetened Beverage, Sugar[Title/Abstract])) OR (Sweetened Beverages, Sugar[Title/Abstract])) OR (Sugar-Added Beverage[Title/Abstract])) OR (Beverage, Sugar-Added[Title/Abstract])) OR (Beverages, Sugar-Added[Title/Abstract])) OR (Sugar Added Beverage[Title/Abstract])) OR (Sweetened Drinks[Title/Abstract])) OR (Sweetened Drink[Title/Abstract])) OR

(Drink, Sweetened[Title/Abstract])) OR (Drinks, Sweetened[Title/Abstract])) OR (Sugar-Sweetened Soft Drinks[Title/Abstract])) OR (Sugar Sweetened Soft Drinks[Title/Abstract])) OR (Sugar-Sweetened Soft Drink[Title/Abstract])) OR (Drink, Sugar-Sweetened Soft[Title/Abstract])) OR (Drinks, Sugar-Sweetened Soft[Title/Abstract])) OR (Soft Drink, Sugar-Sweetened[Title/Abstract])) OR (Soft Drinks, Sugar-Sweetened[Title/Abstract])) OR (Sugar Sweetened Soft Drink[Title/Abstract])) OR (Sweetened Beverages[Title/Abstract])) OR (Sweetened Beverage[Title/Abstract])) OR (Beverage, Sweetened[Title/Abstract])) OR (Beverages, Sweetened[Title/Abstract])) OR (Sugar-Sweetened Sodas[Title/Abstract])) OR (Sugar Sweetened Sodas[Title/Abstract])) OR (Sugar-Sweetened Soda[Title/Abstract])) OR (Soda, Sugar-Sweetened[Title/Abstract])) OR (Sodas, Sugar-Sweetened[Title/Abstract])) OR (Sugar Sweetened Soda[Title/Abstract]))————1

Rheumatoid Arthritis and SSBs

((Rheumatoid Arthritis[Title/Abstract]) OR (Rheumatoid Arthritis[MeSH Terms])) AND (((((((((((((((((((((((((((((((((((((((Sugar-Sweetened Beverages[MeSH Terms]) OR (Sugar-Sweetened Beverages[Title/Abstract])) OR (Beverage, Sugar-Sweetened[Title/Abstract])) OR (Beverages, Sugar-Sweetened[Title/Abstract])) OR (Sugar-Sweetened Beverage[Title/Abstract])) OR (Sugar-Added Beverages[Title/Abstract])) OR (Sugar Added Beverages[Title/Abstract])) OR (Sugar Sweetened Beverage[Title/Abstract])) OR (Beverage, Sugar Sweetened[Title/Abstract])) OR (Beverages, Sugar Sweetened[Title/Abstract])) OR (Sugar Sweetened Beverages[Title/Abstract])) OR (Sweetened Beverage, Sugar[Title/Abstract])) OR (Sweetened Beverages, Sugar[Title/Abstract])) OR (Sugar-Added Beverage[Title/Abstract])) OR (Beverage, Sugar-Added[Title/Abstract])) OR (Beverages, Sugar-Added[Title/Abstract])) OR (Sugar Added Beverage[Title/Abstract])) OR (Sweetened Drinks[Title/Abstract])) OR (Sweetened Drink[Title/Abstract])) OR (Drink, Sweetened[Title/Abstract])) OR (Drinks, Sweetened[Title/Abstract])) OR (Sugar-Sweetened Soft Drinks[Title/Abstract])) OR (Sugar Sweetened Soft Drinks[Title/Abstract])) OR (Sugar-Sweetened Soft Drink[Title/Abstract])) OR (Drink, Sugar-Sweetened Soft[Title/Abstract])) OR (Drinks, Sugar-Sweetened Soft[Title/Abstract])) OR (Soft Drink, Sugar-Sweetened[Title/Abstract])) OR (Soft Drinks, Sugar-Sweetened[Title/Abstract])) OR (Sugar Sweetened Soft Drink[Title/Abstract])) OR (Sweetened Beverages[Title/Abstract])) OR (Sweetened Beverage[Title/Abstract])) OR (Beverage, Sweetened[Title/Abstract])) OR (Beverages, Sweetened[Title/Abstract])) OR (Sugar-Sweetened Sodas[Title/Abstract])) OR (Sugar Sweetened Sodas[Title/Abstract])) OR (Sugar-Sweetened Soda[Title/Abstract])) OR (Soda, Sugar-Sweetened[Title/Abstract])) OR (Sodas, Sugar-Sweetened[Title/Abstract])) OR (Sugar Sweetened Soda[Title/Abstract]))————11

Osteoporosis and SSBs

(((((((((((((((((((((((Osteoporosis[MeSH Terms]) OR (Osteoporosis[Title/Abstract])) OR (Osteoporoses[Title/Abstract])) OR (Osteoporosis, Post-Traumatic[Title/Abstract])) OR (Osteoporosis, Post Traumatic[Title/Abstract])) OR (Post-Traumatic Osteoporoses[Title/Abstract])) OR (Post-Traumatic Osteoporosis[Title/Abstract])) OR (Osteoporosis, Senile[Title/Abstract])) OR (Osteoporoses, Senile[Title/Abstract])) OR (Senile Osteoporoses[Title/Abstract])) OR (Osteoporosis, Involutional[Title/Abstract])) OR (Senile Osteoporosis[Title/Abstract])) OR (Osteoporosis, Age-Related[Title/Abstract])) OR (Osteoporosis, Age Related[Title/Abstract])) OR (Bone Loss, Age-Related[Title/Abstract])) OR (Age-Related Bone Loss[Title/Abstract])) OR (Age-Related Bone Losses[Title/Abstract])) OR (Bone Loss, Age Related[Title/Abstract])) OR (Bone Losses, Age-Related[Title/Abstract])) OR (Age-Related Osteoporosis[Title/Abstract])) OR (Age Related Osteoporosis[Title/Abstract])) OR (Age-Related Osteoporoses[Title/Abstract])) OR (Osteoporoses, Age-Related[Title/Abstract])) AND (((((((((((((((((((((((((((((((((((((((Sugar-Sweetened Beverages[MeSH Terms]) OR (Sugar-Sweetened Beverages[Title/Abstract])) OR (Beverage, Sugar-Sweetened[Title/Abstract])) OR (Beverages, Sugar-Sweetened[Title/Abstract])) OR (Sugar-Sweetened Beverage[Title/Abstract])) OR (Sugar-Added Beverages[Title/Abstract])) OR (Sugar Added Beverages[Title/Abstract])) OR (Sugar Sweetened Beverage[Title/Abstract])) OR (Beverage, Sugar Sweetened[Title/Abstract])) OR (Beverages, Sugar Sweetened[Title/Abstract])) OR (Sugar Sweetened Beverages[Title/Abstract])) OR (Sweetened Beverage, Sugar[Title/Abstract])) OR (Sweetened Beverages, Sugar[Title/Abstract])) OR (Sugar-Added Beverage[Title/Abstract])) OR (Beverage, Sugar-Added[Title/Abstract])) OR (Beverages, Sugar-Added[Title/Abstract])) OR (Sugar Added Beverage[Title/Abstract])) OR

(Sweetened Drinks[Title/Abstract])) OR (Sweetened Drink[Title/Abstract])) OR (Drink, Sweetened[Title/Abstract])) OR (Drinks, Sweetened[Title/Abstract])) OR (Sugar-Sweetened Soft Drinks[Title/Abstract])) OR (Sugar Sweetened Soft Drinks[Title/Abstract])) OR (Sugar-Sweetened Soft Drink[Title/Abstract])) OR (Drink, Sugar-Sweetened Soft[Title/Abstract])) OR (Drinks, Sugar-Sweetened Soft[Title/Abstract])) OR (Soft Drink, Sugar-Sweetened[Title/Abstract])) OR (Soft Drinks, Sugar-Sweetened[Title/Abstract])) OR (Sugar Sweetened Soft Drink[Title/Abstract])) OR (Sweetened Beverages[Title/Abstract])) OR (Sweetened Beverage[Title/Abstract])) OR (Beverage, Sweetened[Title/Abstract])) OR (Beverages, Sweetened[Title/Abstract])) OR (Sugar-Sweetened Sodas[Title/Abstract])) OR (Sugar Sweetened Sodas[Title/Abstract])) OR (Sugar-Sweetened Soda[Title/Abstract])) OR (Soda, Sugar-Sweetened[Title/Abstract])) OR (Sodas, Sugar-Sweetened[Title/Abstract])) OR (Sugar Sweetened Soda[Title/Abstract]))——————8

Gout and alcohol

(((gout[Title/Abstract]) OR (gout[MeSH Terms])) OR (gouts[Title/Abstract])) AND (((("Alcoholic Beverages"[Mesh]) OR (Alcoholic Beverage[Title/Abstract])) OR (Beverage, Alcoholic[Title/Abstract])) OR (Beverages, Alcoholic[Title/Abstract])) ——85

Osteoarthritis and alcohol

(((((((((((Osteoarthritis[Title/Abstract]) OR (Osteoarthritis[MeSH Terms])) OR (Osteoarthritides[Title/Abstract])) OR (Osteoarthrosis[Title/Abstract])) OR (Osteoarthroses[Title/Abstract])) OR (Arthritis, Degenerative[Title/Abstract])) OR (Arthritides, Degenerative[Title/Abstract])) OR (Degenerative Arthritides[Title/Abstract])) OR (Degenerative Arthritis[Title/Abstract])) OR (Arthrosis[Title/Abstract])) OR (Arthroses[Title/Abstract])) OR (Osteoarthrosis Deformans[Title/Abstract])) AND (((("Alcoholic Beverages"[Mesh]) OR (Alcoholic Beverage[Title/Abstract])) OR (Beverage, Alcoholic[Title/Abstract])) OR (Beverages, Alcoholic[Title/Abstract])) ——8

Rheumatoid Arthritis and alcohol

((Rheumatoid Arthritis[Title/Abstract]) OR (Rheumatoid Arthritis[MeSH Terms])) AND (((("Alcoholic Beverages"[Mesh]) OR (Alcoholic Beverage[Title/Abstract])) OR (Beverage, Alcoholic[Title/Abstract])) OR (Beverages, Alcoholic[Title/Abstract])) ——27

Osteoporosis and alcohol

((((((((((((((((((((Osteoporosis[MeSH Terms]) OR (Osteoporosis[Title/Abstract])) OR (Osteoporoses[Title/Abstract])) OR (Osteoporosis, Post-Traumatic[Title/Abstract])) OR (Osteoporosis, Post Traumatic[Title/Abstract])) OR (Post-Traumatic Osteoporoses[Title/Abstract])) OR (Post-Traumatic Osteoporosis[Title/Abstract])) OR (Osteoporosis, Senile[Title/Abstract])) OR (Osteoporoses, Senile[Title/Abstract])) OR (Senile Osteoporoses[Title/Abstract])) OR (Osteoporosis, Involutional[Title/Abstract])) OR (Senile Osteoporosis[Title/Abstract])) OR (Osteoporosis, Age-Related[Title/Abstract])) OR (Osteoporosis, Age Related[Title/Abstract])) OR (Bone Loss, Age-Related[Title/Abstract])) OR (Age-Related Bone Loss[Title/Abstract])) OR (Age-Related Bone Losses[Title/Abstract])) OR (Bone Loss, Age Related[Title/Abstract])) OR (Bone Losses, Age-Related[Title/Abstract])) OR (Age-Related Osteoporosis[Title/Abstract])) OR (Age Related Osteoporosis[Title/Abstract])) OR (Age-Related Osteoporoses[Title/Abstract])) OR (Osteoporoses, Age-Related[Title/Abstract])) AND (((("Alcoholic Beverages"[Mesh]) OR (Alcoholic Beverage[Title/Abstract])) OR (Beverage, Alcoholic[Title/Abstract])) OR (Beverages, Alcoholic[Title/Abstract])) ——65

Cochrane

#1 MeSH descriptor: [Gout] explode all trees 587

#2 (gout):ti,ab,kw (Word variations have been searched) 1995

#3 (gouts):ti,ab,kw (Word variations have been searched) 1995

#4 #1 OR #2 OR #3 2022

#5 MeSH descriptor: [Osteoarthritis] explode all trees 10785

#6 (Osteoarthritis):ti,ab,kw (Word variations have been searched) 23978

#7 (Osteoarthritides):ti,ab,kw (Word variations have been searched) 1

#8 (Arthritis, Degenerative):ti,ab,kw (Word variations have been searched) 368

#9 (Arthritides, Degenerative):ti,ab,kw (Word variations have been searched) 0

#10 (Degenerative Arthritides):ti,ab,kw (Word variations have been searched) 0

#11 (Degenerative Arthritis):ti,ab,kw (Word variations have been searched) 368

#12 (Arthrosis):ti,ab,kw (Word variations have been searched) 722

#13 (Arthroses):ti,ab,kw (Word variations have been searched) 62

#14 (Osteoarthrosis Deformans):ti,ab,kw (Word variations have been searched) 6

#15 #5 OR #6 OR #7 OR #8 OR #9 OR #10 OR #11 OR #12 OR #13 OR #14 24372

#16 MeSH descriptor: [Arthritis, Rheumatoid] explode all trees 8026

#17 (Rheumatoid Arthritis):ti,ab,kw (Word variations have been searched) 18964

#18 #16 OR #17 19384

#19 MeSH descriptor: [Osteoporosis] explode all trees 5476

#20 (Osteoporosis):ti,ab,kw (Word variations have been searched) 12306

#21 (Osteoporoses):ti,ab,kw (Word variations have been searched) 12

#22 (Osteoporosis, Post-Traumatic):ti,ab,kw (Word variations have been searched) 10

#23 (Osteoporosis, Post Traumatic):ti,ab,kw (Word variations have been searched) 20

#24 (Post-Traumatic Osteoporoses):ti,ab,kw (Word variations have been searched) 0

#25 (Post-Traumatic Osteoporosis):ti,ab,kw (Word variations have been searched) 10

#26 (Osteoporosis, Senile):ti,ab,kw (Word variations have been searched) 114

#27 (Osteoporoses, Senile):ti,ab,kw (Word variations have been searched) 0

#28 (Senile Osteoporoses):ti,ab,kw (Word variations have been searched) 0

#29 (Osteoporosis, Involutional):ti,ab,kw (Word variations have been searched) 54

#30 (Senile Osteoporosis):ti,ab,kw (Word variations have been searched) 114

#31 (Osteoporosis, Age-Related):ti,ab,kw (Word variations have been searched) 175

#32 (Osteoporosis, Age Related):ti,ab,kw (Word variations have been searched) 2135

#33 (Bone Loss, Age-Related):ti,ab,kw (Word variations have been searched) 136

#34 (Age-Related Bone Loss):ti,ab,kw (Word variations have been searched) 136

#35 (Age-Related Bone Losses):ti,ab,kw (Word variations have been searched) 136

#36 (Bone Loss, Age Related):ti,ab,kw (Word variations have been searched) 1651

#37 (Bone Losses, Age-Related):ti,ab,kw (Word variations have been searched) 136

#38 (Age-Related Osteoporosis):ti,ab,kw (Word variations have been searched) 175

#39 (Age Related Osteoporosis):ti,ab,kw (Word variations have been searched) 2135

#40 (Age-Related Osteoporoses):ti,ab,kw (Word variations have been searched) 0

#41 (Osteoporoses, Age-Related):ti,ab,kw (Word variations have been searched) 0

#42 #19 OR #20 OR #21 OR #22 OR #23 OR #24 OR #25 OR #26 OR #27 OR #28 OR #29 OR #30 OR #31 OR #32 OR #33 OR #34 OR #35 OR #36 OR #37 OR #38 OR #39 OR #40 OR #41 13509

#43 MeSH descriptor: [Tea] explode all trees 707

#44 (Tea):ti,ab,kw (Word variations have been searched) 4864

#45 (Black Tea):ti,ab,kw (Word variations have been searched) 359

#46 (Black Teas):ti,ab,kw (Word variations have been searched) 359

#47 (Tea, Black):ti,ab,kw (Word variations have been searched) 359

#48 (Green Tea):ti,ab,kw (Word variations have been searched) 1479

#49 (Green Teas):ti,ab,kw (Word variations have been searched) 1479

#50 (Tea,Green):ti,ab,kw (Word variations have been searched)28

#51 (Teas,Green):ti,ab,kw (Word variations have been searched) 28

#52 #43 OR #44 OR #45 OR #46 OR #47 OR #48 OR #49 #50 OR #51 4864

#53 MeSH descriptor: [Coffee] explode all trees 545

#54 (Coffee):ti,ab,kw (Word variations have been searched) 2209

#55 #53 OR #54 2209

#56 MeSH descriptor: [Sugar-Sweetened Beverages] explode all trees106

#57 (Sugar-Sweetened Beverages):ti,ab,kw (Word variations have been searched) 814

#58 (Beverage, Sugar-Sweetened):ti,ab,kw (Word variations have been searched) 814

#59 (Beverages, Sugar-Sweetened):ti,ab,kw (Word variations have been searched) 814

#60 (Sugar-Sweetened Beverage):ti,ab,kw (Word variations have been searched) 814

#61 (Sugar-Added Beverages):ti,ab,kw (Word variations have been searched) 13

#62 (Sugar Added Beverages):ti,ab,kw (Word variations have been searched) 320

#63 (Sugar Sweetened Beverage):ti,ab,kw (Word variations have been searched) 962

#64 (Beverage, Sugar Sweetened):ti,ab,kw (Word variations have been searched) 962

#65 (Beverages, Sugar Sweetened):ti,ab,kw (Word variations have been searched) 962

#66 (Sugar Sweetened Beverages):ti,ab,kw (Word variations have been searched) 962

#67 (Sweetened Beverage, Sugar):ti,ab,kw (Word variations have been searched) 962

#68 (Sweetened Beverages, Sugar):ti,ab,kw (Word variations have been searched) 962

#69 (Sugar-Added Beverage):ti,ab,kw (Word variations have been searched)13

#70 (Beverage, Sugar-Added):ti,ab,kw (Word variations have been searched) 13

#71 (Beverages, Sugar-Added):ti,ab,kw (Word variations have been searched) 13

#72 (Sugar Added Beverage):ti,ab,kw (Word variations have been searched) 320

#73 (Sweetened Drinks):ti,ab,kw (Word variations have been searched) 700

#74 (Sweetened Drink):ti,ab,kw (Word variations have been searched) 700

#75 (Drink, Sweetened):ti,ab,kw (Word variations have been searched) 700

#76 (Drinks, Sweetened):ti,ab,kw (Word variations have been searched) 700

#77 (Sugar-Sweetened Soft Drinks):ti,ab,kw (Word variations have been searched) 87

#78 (Sugar Sweetened Soft Drinks):ti,ab,kw (Word variations have been searched) 110

#79 (Sugar-Sweetened Soft Drink):ti,ab,kw (Word variations have been searched) 87

#80 (Drink, Sugar-Sweetened Soft):ti,ab,kw (Word variations have been searched) 87

#81 (Drinks, Sugar-Sweetened Soft):ti,ab,kw (Word variations have been searched) 87

#82 (Soft Drink, Sugar-Sweetened):ti,ab,kw (Word variations have been searched) 87

#83 (Soft Drinks, Sugar-Sweetened):ti,ab,kw (Word variations have been searched) 87

#84 (Sugar Sweetened Soft Drink):ti,ab,kw (Word variations have been searched) 110

#85 (Sweetened Beverages):ti,ab,kw (Word variations have been searched) 1308

#86 (Sweetened Beverage):ti,ab,kw (Word variations have been searched) 1308

#87 (Beverage, Sweetened):ti,ab,kw (Word variations have been searched) 1308

#88 (Beverages, Sweetened):ti,ab,kw (Word variations have been searched) 1308

#89 (Sugar-Sweetened Sodas):ti,ab,kw (Word variations have been searched) 58

#90 (Sugar Sweetened Sodas):ti,ab,kw (Word variations have been searched) 67

#91 (Sugar-Sweetened Soda):ti,ab,kw (Word variations have been searched) 58

#92 (Soda, Sugar-Sweetened):ti,ab,kw (Word variations have been searched) 58

#93 (Sodas, Sugar-Sweetened):ti,ab,kw (Word variations have been searched) 58

#94 (Sugar Sweetened Soda):ti,ab,kw (Word variations have been searched) 67

#95 #56 OR #57 OR #58 OR #59 OR #60 OR #61 OR #62 OR #63 OR #64 OR #65 OR #66 OR # 67 OR #68 OR #69 OR #70 OR #71 OR #72 OR #73 OR #74 OR #75 OR #76 OR #77 OR #78 OR #79 OR #80 OR #81 OR #82 OR #83 OR #84 OR #85 OR #86 OR #87 OR #88 OR #89 OR #90 OR #91 OR #92 OR #93 OR #94 98427

#96 #4 AND #52 10

#97 #15 AND #52 47

#98 #18 AND #52 22

#99 #42 AND #52 19

#100 #4 AND #55 2

#101 #15 AND #55 3

#102 #18 AND #55 12

#103 #42 AND #55 10

#104 #4 AND #96 10

#105 #15 AND #96 0

#106 #18 AND #96 0

#107 #42 AND #96 0

#1 (Alcoholic Beverage):ti,ab,kw (Word variations have been searched) 2006

#2 (Beverage, Alcoholic):ti,ab,kw (Word variations have been searched) 2006

#3 (Beverages, Alcoholic):ti,ab,kw (Word variations have been searched) 2007

#4 MeSH descriptor: [Alcoholic Beverages] explode all trees 722

#5 #1 OR #2 OR #3 OR #4 2295

#6 MeSH descriptor: [Gout] explode all trees 626

#7 (gout):ti,ab,kw (Word variations have been searched) 2154

#8 (gouts):ti,ab,kw (Word variations have been searched) 5

#9 #6 OR #7 OR #8 2184

#10 MeSH descriptor: [Osteoarthritis] explode all trees 11267

#11 (Osteoarthritis):ti,ab,kw (Word variations have been searched) 25849

#12 (Osteoarthritides):ti,ab,kw (Word variations have been searched) 1

#13 (Arthritis, Degenerative):ti,ab,kw (Word variations have been searched) 404

#14 (Arthritides, Degenerative):ti,ab,kw (Word variations have been searched) 0

#15 (Degenerative Arthritides):ti,ab,kw (Word variations have been searched) 0

#16 (Degenerative Arthritis):ti,ab,kw (Word variations have been searched) 404

#17 (Arthrosis):ti,ab,kw (Word variations have been searched) 769

#18 (Arthroses):ti,ab,kw (Word variations have been searched) 46

#19 (Osteoarthrosis Deformans):ti,ab,kw (Word variations have been searched) 6

#20 #10 OR #11 OR #12 OR #13 OR #14 OR #15 OR #16 OR #17 OR #18 OR #19 26259

#21 MeSH descriptor: [Arthritis, Rheumatoid] explode all trees 8194

#22 (Rheumatoid Arthritis):ti,ab,kw (Word variations have been searched) 19640

#23 #21 OR #22 20083

#24 MeSH descriptor: [Osteoporosis] explode all trees 5559

#25 (Osteoporosis):ti,ab,kw (Word variations have been searched) 12713

#26 (Osteoporoses):ti,ab,kw (Word variations have been searched) 4

#27 (Osteoporosis, Post-Traumatic):ti,ab,kw (Word variations have been searched) 10

#28 (Osteoporosis, Post Traumatic):ti,ab,kw (Word variations have been searched) 20

#29 (Post-Traumatic Osteoporoses):ti,ab,kw (Word variations have been searched) 0

#30 (Post-Traumatic Osteoporosis):ti,ab,kw (Word variations have been searched) 10

#31 (Osteoporosis, Senile):ti,ab,kw (Word variations have been searched) 117

#32 (Osteoporoses, Senile):ti,ab,kw (Word variations have been searched) 0

#33 (Senile Osteoporoses):ti,ab,kw (Word variations have been searched) 0

#34 (Osteoporosis, Involutional):ti,ab,kw (Word variations have been searched) 50

#35 (Senile Osteoporosis):ti,ab,kw (Word variations have been searched) 117

#36 (Osteoporosis, Age-Related):ti,ab,kw (Word variations have been searched) 183

#37 (Osteoporosis, Age Related):ti,ab,kw (Word variations have been searched) 816

#38 (Bone Loss, Age-Related):ti,ab,kw (Word variations have been searched) 136

#39 (Age-Related Bone Loss):ti,ab,kw (Word variations have been searched) 136

#40 (Age-Related Bone Losses):ti,ab,kw (Word variations have been searched) 10

#41 (Bone Loss, Age Related):ti,ab,kw (Word variations have been searched) 632

#42 (Bone Losses, Age-Related):ti,ab,kw (Word variations have been searched) 10

#43 (Age-Related Osteoporosis):ti,ab,kw (Word variations have been searched) 183

#44 (Age Related Osteoporosis):ti,ab,kw (Word variations have been searched) 816

#45 (Age-Related Osteoporoses):ti,ab,kw (Word variations have been searched) 0

#46 (Osteoporoses, Age-Related):ti,ab,kw (Word variations have been searched) 0

#47 #24 OR #25 OR #26 OR #27 OR #28 OR #29 OR #30 OR #31 OR #32 OR #33 OR #34 OR #35 OR #36 OR #37 OR #38 OR #39 OR #40 OR #41 OR #42 OR #43 OR #44 OR #45 OR #46 13171

#48 #5 AND #9 6

#49 #5 AND #23 3

#50 #5 AND #20 4

#51 #5 AND #47 6

Embase

No. Query Results Results Date

#97. (('gout'/exp OR 'gout':ab,ti OR 'gouts':ab,ti) AND ('tea'/exp OR 'tea':ab,ti OR 'black tea':ab,ti OR 'black teas':ab,ti OR 'tea,black':ab,ti OR 'teas,black':ab,ti OR 'green tea':ab,ti OR 'green teas':ab,ti OR 'tea,green':ab,ti OR 'teas,green':ab,ti)) OR (('gout'/exp OR 'gout':ab,ti OR 'gouts':ab,ti) AND ('coffee'/exp OR 'coffee':ab,ti)) OR (('gout'/exp OR 'gout':ab,ti OR 'gouts':ab,ti) AND ('sugar-sweetened beverage'/exp OR 'sugar-sweetened beverages':ab,ti OR 'sugar-sweetened beverage':ab,ti OR 'beverage, sugar-sweetened':ab,ti OR 'beverages, sugar-sweetened':ab,ti OR 'sugar-added beverages':ab,ti OR 'sugar added beverages':ab,ti OR 'sugar sweetened beverage':ab,ti OR 'beverage, sugar sweetened':ab,ti OR 'beverages, sugar **1,524** **21 May 2024**

sweetened':ab,ti OR 'sugar sweetened beverages':ab,ti OR 'sweetened beverage, sugar':ab,ti OR 'sweetened beverages, sugar':ab,ti OR 'sugar-added beverage':ab,ti OR 'beverages, sugar-added':ab,ti OR 'sugar added beverage':ab,ti OR 'sweetened drinks':ab,ti OR 'sweetened drink':ab,ti OR 'drink, sweetened':ab,ti OR 'drinks, sweetened':ab,ti OR 'sugar-sweetened soft drinks':ab,ti OR 'sugar sweetened soft drinks':ab,ti OR 'sugar-sweetened soft drink':ab,ti OR 'soft drink, sugar-sweetened':ab,ti OR 'soft drinks, sugar-sweetened':ab,ti OR 'sugar sweetened soft drink':ab,ti OR 'sweetened beverages':ab,ti OR 'sweetened beverage':ab,ti OR 'beverage, sweetened':ab,ti OR 'beverages, sweetened':ab,ti OR 'sugar-sweetened sodas':ab,ti OR 'sugar sweetened sodas':ab,ti OR 'sugar-sweetened soda':ab,ti OR 'soda, sugar-sweetened':ab,ti OR 'sugar sweetened soda':ab,ti)) OR (('osteoarthritis'/exp OR 'osteoarthritis':ab,ti OR 'osteoarthritides':ab,ti OR 'osteoarthrosis':ab,ti OR 'osteoarthroses':ab,ti OR 'arthrosis':ab,ti OR 'arthroses':ab,ti) AND ('tea'/exp OR 'tea':ab,ti OR 'black tea':ab,ti OR 'black teas':ab,ti OR 'tea,black':ab,ti OR 'teas,black':ab,ti OR 'green tea':ab,ti OR 'green teas':ab,ti OR 'tea,green':ab,ti OR 'teas,green':ab,ti)) OR (('osteoarthritis'/exp OR 'osteoarthritis':ab,ti OR 'osteoarthritides':ab,ti OR 'osteoarthrosis':ab,ti OR 'osteoarthroses':ab,ti OR 'arthrosis':ab,ti OR 'arthroses':ab,ti) AND ('coffee'/exp OR 'coffee':ab,ti)) OR (('osteoarthritis'/exp OR 'osteoarthritis':ab,ti OR 'osteoarthritides':ab,ti OR 'osteoarthrosis':ab,ti OR 'osteoarthroses':ab,ti OR 'arthrosis':ab,ti OR 'arthroses':ab,ti) AND ('sugar-sweetened beverage'/exp OR 'sugar-sweetened beverages':ab,ti OR 'sugar-sweetened beverage':ab,ti OR 'beverage, sugar-sweetened':ab,ti OR 'beverages, sugar-sweetened':ab,ti OR 'sugar-added beverages':ab,ti OR 'sugar added beverages':ab,ti

OR 'sugar sweetened beverage':ab,ti OR 'beverage, sugar sweetened':ab,ti OR 'beverages, sugar sweetened':ab,ti OR 'sugar sweetened beverages':ab,ti OR 'sweetened beverage, sugar':ab,ti OR 'sweetened beverages, sugar':ab,ti OR 'sugar-added beverage':ab,ti OR 'beverages, sugar-added':ab,ti OR 'sugar added beverage':ab,ti OR 'sweetened drinks':ab,ti OR 'sweetened drink':ab,ti OR 'drink, sweetened':ab,ti OR 'drinks, sweetened':ab,ti OR 'sugar-sweetened soft drinks':ab,ti OR 'sugar sweetened soft drinks':ab,ti OR 'sugar-sweetened soft drink':ab,ti OR 'soft drink, sugar-sweetened':ab,ti OR 'soft drinks, sugar-sweetened':ab,ti OR 'sugar sweetened soft drink':ab,ti OR 'sweetened beverages':ab,ti OR 'sweetened beverage':ab,ti OR 'beverage, sweetened':ab,ti OR 'beverages, sweetened':ab,ti OR 'sugar-sweetened sodas':ab,ti OR 'sugar sweetened sodas':ab,ti OR 'sugar-sweetened soda':ab,ti OR 'soda, sugar-sweetened':ab,ti OR 'sugar sweetened soda':ab,ti)) OR (('rheumatoid arthritis'/exp OR 'rheumatoid arthritis':ab,ti) AND ('tea'/exp OR 'tea':ab,ti OR 'black tea':ab,ti OR 'black teas':ab,ti OR 'tea,black':ab,ti OR 'teas,black':ab,ti OR 'green tea':ab,ti OR 'green teas':ab,ti OR 'tea,green':ab,ti OR 'teas,green':ab,ti)) OR (('rheumatoid arthritis'/exp OR 'rheumatoid arthritis':ab,ti) AND ('coffee'/exp OR 'coffee':ab,ti)) OR (('rheumatoid arthritis'/exp OR 'rheumatoid arthritis':ab,ti) AND ('sugar-sweetened beverage'/exp OR 'sugar-sweetened beverages':ab,ti OR 'sugar-sweetened beverage':ab,ti OR 'beverage, sugar-sweetened':ab,ti OR 'beverages, sugar-sweetened':ab,ti OR 'sugar-added beverages':ab,ti OR 'sugar added beverages':ab,ti OR 'sugar sweetened beverage':ab,ti OR 'beverage, sugar sweetened':ab,ti OR 'beverages, sugar sweetened':ab,ti OR 'sugar sweetened beverages':ab,ti OR 'sweetened beverage, sugar':ab,ti OR 'sweetened beverages, sugar':ab,ti OR 'sugar-added beverage':ab,ti OR

'beverages, sugar-added':ab,ti OR 'sugar added beverage':ab,ti OR 'sweetened drinks':ab,ti OR 'sweetened drink':ab,ti OR 'drink, sweetened':ab,ti OR 'drinks, sweetened':ab,ti OR 'sugar-sweetened soft drinks':ab,ti OR 'sugar sweetened soft drinks':ab,ti OR 'sugar-sweetened soft drink':ab,ti OR 'soft drink, sugar-sweetened':ab,ti OR 'soft drinks, sugar-sweetened':ab,ti OR 'sugar sweetened soft drink':ab,ti OR 'sweetened beverages':ab,ti OR 'sweetened beverage':ab,ti OR 'beverage, sweetened':ab,ti OR 'beverages, sweetened':ab,ti OR 'sugar-sweetened sodas':ab,ti OR 'sugar sweetened sodas':ab,ti OR 'sugar-sweetened soda':ab,ti OR 'soda, sugar-sweetened':ab,ti OR 'sugar sweetened soda':ab,ti)) OR (('osteoporosis'/exp OR 'osteoporosis':ab,ti OR 'osteoporoses':ab,ti OR 'osteoporosis, post-traumatic':ab,ti OR 'osteoporosis, post traumatic':ab,ti OR 'post-traumatic osteoporosis':ab,ti OR 'osteoporosis, senile':ab,ti OR 'senile osteoporoses':ab,ti OR 'senile osteoporosis':ab,ti OR 'osteoporosis, age-related':ab,ti OR 'osteoporosis, age related':ab,ti OR 'bone loss, age-related':ab,ti OR 'age-related bone loss':ab,ti OR 'age-related bone losses':ab,ti OR 'bone loss, age related':ab,ti OR 'age-related osteoporosis':ab,ti OR 'age related osteoporosis':ab,ti OR 'age-related osteoporoses':ab,ti) AND ('tea'/exp OR 'tea':ab,ti OR 'black tea':ab,ti OR 'black teas':ab,ti OR 'tea,black':ab,ti OR 'teas,black':ab,ti OR 'green tea':ab,ti OR 'green teas':ab,ti OR 'tea,green':ab,ti OR 'teas,green':ab,ti)) OR (('osteoporosis'/exp OR 'osteoporosis':ab,ti OR 'osteoporoses':ab,ti OR 'osteoporosis, post-traumatic':ab,ti OR 'osteoporosis, post traumatic':ab,ti OR 'post-traumatic osteoporosis':ab,ti OR 'osteoporosis, senile':ab,ti OR 'senile osteoporoses':ab,ti OR 'senile osteoporosis':ab,ti OR 'osteoporosis, age-related':ab,ti OR 'osteoporosis, age

related':ab,ti OR 'bone loss, age-related':ab,ti OR 'age-related bone loss':ab,ti OR 'age-related bone losses':ab,ti OR 'bone loss, age related':ab,ti OR 'age-related osteoporosis':ab,ti OR 'age related osteoporosis':ab,ti OR 'age-related osteoporoses':ab,ti) AND ('coffee'/exp OR 'coffee':ab,ti)) OR (('osteoporosis'/exp OR 'osteoporosis':ab,ti OR 'osteoporoses':ab,ti OR 'osteoporosis, post-traumatic':ab,ti OR 'osteoporosis, post traumatic':ab,ti OR 'post-traumatic osteoporosis':ab,ti OR 'osteoporosis, senile':ab,ti OR 'senile osteoporoses':ab,ti OR 'senile osteoporosis':ab,ti OR 'osteoporosis, age-related':ab,ti OR 'osteoporosis, age related':ab,ti OR 'bone loss, age-related':ab,ti OR 'age-related bone loss':ab,ti OR 'age-related bone losses':ab,ti OR 'bone loss, age related':ab,ti OR 'age-related osteoporosis':ab,ti OR 'age related osteoporosis':ab,ti OR 'age-related osteoporoses':ab,ti) AND ('sugar-sweetened beverage'/exp OR 'sugar-sweetened beverages':ab,ti OR 'sugar-sweetened beverage':ab,ti OR 'beverage, sugar-sweetened':ab,ti OR 'beverages, sugar-sweetened':ab,ti OR 'sugar-added beverages':ab,ti OR 'sugar added beverages':ab,ti OR 'sugar sweetened beverage':ab,ti OR 'beverage, sugar sweetened':ab,ti OR 'beverages, sugar sweetened':ab,ti OR 'sugar sweetened beverages':ab,ti OR 'sweetened beverage, sugar':ab,ti OR 'sweetened beverages, sugar':ab,ti OR 'sugar-added beverage':ab,ti OR 'beverages, sugar-added':ab,ti OR 'sugar added beverage':ab,ti OR 'sweetened drinks':ab,ti OR 'sweetened drink':ab,ti OR 'drink, sweetened':ab,ti OR 'drinks, sweetened':ab,ti OR 'sugar-sweetened soft drinks':ab,ti OR 'sugar sweetened soft drinks':ab,ti OR 'sugar-sweetened soft drink':ab,ti OR 'soft drink, sugar-sweetened':ab,ti OR 'soft drinks, sugar-sweetened':ab,ti OR 'sugar sweetened soft

drink':ab,ti OR 'sweetened beverages':ab,ti OR 'sweetened beverage':ab,ti OR 'beverage, sweetened':ab,ti OR 'beverages, sweetened':ab,ti OR 'sugar-sweetened sodas':ab,ti OR 'sugar sweetened sodas':ab,ti OR 'sugar-sweetened soda':ab,ti OR 'soda, sugar-sweetened':ab,ti OR 'sugar sweetened soda':ab,ti))

#96.	('osteoporosis'/exp OR 'osteoporosis':ab,ti OR 'osteoporoses':ab,ti OR 'osteoporosis, post-traumatic':ab,ti OR 'osteoporosis, post traumatic':ab,ti OR 'post-traumatic osteoporosis':ab,ti OR 'osteoporosis, senile':ab,ti OR 'senile osteoporoses':ab,ti OR 'senile osteoporosis':ab,ti OR 'osteoporosis, age-related':ab,ti OR 'osteoporosis, age related':ab,ti OR 'bone loss, age-related':ab,ti OR 'age-related bone loss':ab,ti OR 'age-related bone losses':ab,ti OR 'bone loss, age related':ab,ti OR 'age-related osteoporosis':ab,ti OR 'age related osteoporosis':ab,ti OR 'age-related osteoporoses':ab,ti) AND ('sugar-sweetened beverage'/exp OR 'sugar-sweetened beverages':ab,ti OR 'sugar-sweetened beverage':ab,ti OR 'beverage, sugar-sweetened':ab,ti OR 'beverages, sugar-sweetened':ab,ti OR 'sugar-added beverages':ab,ti OR 'sugar added beverages':ab,ti OR 'sugar sweetened beverage':ab,ti OR 'beverage, sugar sweetened':ab,ti OR 'beverages, sugar sweetened':ab,ti OR 'sugar sweetened beverages':ab,ti OR 'sweetened beverage, sugar':ab,ti OR 'sweetened beverages, sugar':ab,ti OR 'sugar-added beverage':ab,ti OR 'beverages, sugar-added':ab,ti OR 'sugar added beverage':ab,ti OR 'sweetened drinks':ab,ti OR 'sweetened drink':ab,ti OR 'drink, sweetened':ab,ti OR 'drinks, sweetened':ab,ti OR 'sugar-sweetened soft drinks':ab,ti OR 'sugar sweetened soft drinks':ab,ti OR 'sugar-sweetened soft drink':ab,ti OR 'soft drink, sugar-sweetened':ab,ti OR 'soft drinks, sugar-sweetened':ab,ti OR 'sugar sweetened soft drink':ab,ti OR 'sweetened beverages':ab,ti OR	15	21 May 2024

	'sweetened beverage':ab,ti OR 'beverage, sweetened':ab,ti OR 'beverages, sweetened':ab,ti OR 'sugar-sweetened sodas':ab,ti OR 'sugar sweetened sodas':ab,ti OR 'sugar-sweetened soda':ab,ti OR 'soda, sugar-sweetened':ab,ti OR 'sugar sweetened soda':ab,ti)		
#95.	('osteoporosis'/exp OR 'osteoporosis':ab,ti OR 'osteoporoses':ab,ti OR 'osteoporosis, post-traumatic':ab,ti OR 'osteoporosis, post traumatic':ab,ti OR 'post-traumatic osteoporosis':ab,ti OR 'osteoporosis, senile':ab,ti OR 'senile osteoporoses':ab,ti OR 'senile osteoporosis':ab,ti OR 'osteoporosis, age-related':ab,ti OR 'osteoporosis, age related':ab,ti OR 'bone loss, age-related':ab,ti OR 'age-related bone loss':ab,ti OR 'age-related bone losses':ab,ti OR 'bone loss, age related':ab,ti OR 'age-related osteoporosis':ab,ti OR 'age related osteoporosis':ab,ti OR 'age-related osteoporoses':ab,ti) AND ('coffee'/exp OR 'coffee':ab,ti)	305	21 May 2024
#94.	('osteoporosis'/exp OR 'osteoporosis':ab,ti OR 'osteoporoses':ab,ti OR 'osteoporosis, post-traumatic':ab,ti OR 'osteoporosis, post traumatic':ab,ti OR 'post-traumatic osteoporosis':ab,ti OR 'osteoporosis, senile':ab,ti OR 'senile osteoporoses':ab,ti OR 'senile osteoporosis':ab,ti OR 'osteoporosis, age-related':ab,ti OR 'osteoporosis, age related':ab,ti OR 'bone loss, age-related':ab,ti OR 'age-related bone loss':ab,ti OR 'age-related bone losses':ab,ti OR 'bone loss, age related':ab,ti OR 'age-related osteoporosis':ab,ti OR 'age related osteoporosis':ab,ti OR 'age-related osteoporoses':ab,ti) AND ('tea'/exp OR 'tea':ab,ti OR 'black tea':ab,ti OR 'black teas':ab,ti OR 'tea,black':ab,ti OR 'teas,black':ab,ti OR 'green tea':ab,ti OR 'green teas':ab,ti OR 'tea,green':ab,ti OR 'teas,green':ab,ti)	367	21 May 2024
#93.	('rheumatoid arthritis'/exp OR 'rheumatoid arthritis':ab,ti) AND ('sugar-sweetened	23	21 May 2024

	beverage'/exp OR 'sugar-sweetened beverages':ab,ti OR 'sugar-sweetened beverage':ab,ti OR 'beverage, sugar-sweetened':ab,ti OR 'beverages, sugar-sweetened':ab,ti OR 'sugar-added beverages':ab,ti OR 'sugar added beverages':ab,ti OR 'sugar sweetened beverage':ab,ti OR 'beverage, sugar sweetened':ab,ti OR 'beverages, sugar sweetened':ab,ti OR 'sugar sweetened beverages':ab,ti OR 'sweetened beverage, sugar':ab,ti OR 'sweetened beverages, sugar':ab,ti OR 'sugar-added beverage':ab,ti OR 'beverages, sugar-added':ab,ti OR 'sugar added beverage':ab,ti OR 'sweetened drinks':ab,ti OR 'sweetened drink':ab,ti OR 'drink, sweetened':ab,ti OR 'drinks, sweetened':ab,ti OR 'sugar-sweetened soft drinks':ab,ti OR 'sugar sweetened soft drinks':ab,ti OR 'sugar-sweetened soft drink':ab,ti OR 'soft drink, sugar-sweetened':ab,ti OR 'soft drinks, sugar-sweetened':ab,ti OR 'sugar sweetened soft drink':ab,ti OR 'sweetened beverages':ab,ti OR 'sweetened beverage':ab,ti OR 'beverage, sweetened':ab,ti OR 'beverages, sweetened':ab,ti OR 'sugar-sweetened sodas':ab,ti OR 'sugar sweetened sodas':ab,ti OR 'sugar-sweetened soda':ab,ti OR 'soda, sugar-sweetened':ab,ti OR 'sugar sweetened soda':ab,ti)		
#92.	('rheumatoid arthritis'/exp OR 'rheumatoid arthritis':ab,ti) AND ('coffee'/exp OR 'coffee':ab,ti)	137	21 May 2024
#91.	('rheumatoid arthritis'/exp OR 'rheumatoid arthritis':ab,ti) AND ('tea'/exp OR 'tea':ab,ti OR 'black tea':ab,ti OR 'black teas':ab,ti OR 'tea,black':ab,ti OR 'teas,black':ab,ti OR 'green tea':ab,ti OR 'green teas':ab,ti OR 'tea,green':ab,ti OR 'teas,green':ab,ti)	380	21 May 2024
#90.	('osteoarthritis'/exp OR 'osteoarthritis':ab,ti OR 'osteoarthritides':ab,ti OR 'osteoarthrosis':ab,ti OR 'osteoarthroses':ab,ti OR 'arthrosis':ab,ti OR 'arthroses':ab,ti) AND ('sugar-sweetened beverage'/exp OR 'sugar-sweetened beverages':ab,ti OR 'sugar-sweetened beverage':ab,ti OR 'beverage,	15	21 May 2024

sugar-sweetened':ab,ti OR 'beverages, sugar-sweetened':ab,ti OR 'sugar-added beverages':ab,ti OR 'sugar added beverages':ab,ti OR 'sugar sweetened beverage':ab,ti OR 'beverage, sugar sweetened':ab,ti OR 'beverages, sugar sweetened':ab,ti OR 'sugar sweetened beverages':ab,ti OR 'sweetened beverage, sugar':ab,ti OR 'sweetened beverages, sugar':ab,ti OR 'sugar-added beverage':ab,ti OR 'beverages, sugar-added':ab,ti OR 'sugar added beverage':ab,ti OR 'sweetened drinks':ab,ti OR 'sweetened drink':ab,ti OR 'drink, sweetened':ab,ti OR 'drinks, sweetened':ab,ti OR 'sugar-sweetened soft drinks':ab,ti OR 'sugar sweetened soft drinks':ab,ti OR 'sugar-sweetened soft drink':ab,ti OR 'soft drink, sugar-sweetened':ab,ti OR 'soft drinks, sugar-sweetened':ab,ti OR 'sugar sweetened soft drink':ab,ti OR 'sweetened beverages':ab,ti OR 'sweetened beverage':ab,ti OR 'beverage, sweetened':ab,ti OR 'beverages, sweetened':ab,ti OR 'sugar-sweetened sodas':ab,ti OR 'sugar sweetened sodas':ab,ti OR 'sugar-sweetened soda':ab,ti OR 'soda, sugar-sweetened':ab,ti OR 'sugar sweetened soda':ab,ti)

**#89.**	**('osteoarthritis'/exp OR 'osteoarthritis':ab,ti OR 'osteoarthritides':ab,ti OR 'osteoarthrosis':ab,ti OR 'osteoarthroses':ab,ti OR 'arthrosis':ab,ti OR 'arthroses':ab,ti) AND ('coffee'/exp OR 'coffee':ab,ti)**	**78**	**21 May 2024**
**#88.**	**('osteoarthritis'/exp OR 'osteoarthritis':ab,ti OR 'osteoarthritides':ab,ti OR 'osteoarthrosis':ab,ti OR 'osteoarthroses':ab,ti OR 'arthrosis':ab,ti OR 'arthroses':ab,ti) AND ('tea'/exp OR 'tea':ab,ti OR 'black tea':ab,ti OR 'black teas':ab,ti OR 'tea,black':ab,ti OR 'teas,black':ab,ti OR 'green tea':ab,ti OR 'green teas':ab,ti OR 'tea,green':ab,ti OR 'teas,green':ab,ti)**	**327**	**21 May 2024**
**#87.**	**('gout'/exp OR 'gout':ab,ti OR 'gouts':ab,ti) AND ('sugar-sweetened beverage'/exp OR 'sugar-sweetened beverages':ab,ti OR 'sugar-sweetened beverage':ab,ti OR 'beverage, sugar-sweetened':ab,ti OR 'beverages,**	**74**	**21 May 2024**

	sugar-sweetened':ab,ti OR 'sugar-added beverages':ab,ti OR 'sugar added beverages':ab,ti OR 'sugar sweetened beverage':ab,ti OR 'beverage, sugar sweetened':ab,ti OR 'beverages, sugar sweetened':ab,ti OR 'sugar sweetened beverages':ab,ti OR 'sweetened beverage, sugar':ab,ti OR 'sweetened beverages, sugar':ab,ti OR 'sugar-added beverage':ab,ti OR 'beverages, sugar-added':ab,ti OR 'sugar added beverage':ab,ti OR 'sweetened drinks':ab,ti OR 'sweetened drink':ab,ti OR 'drink, sweetened':ab,ti OR 'drinks, sweetened':ab,ti OR 'sugar-sweetened soft drinks':ab,ti OR 'sugar sweetened soft drinks':ab,ti OR 'sugar-sweetened soft drink':ab,ti OR 'soft drink, sugar-sweetened':ab,ti OR 'soft drinks, sugar-sweetened':ab,ti OR 'sugar sweetened soft drink':ab,ti OR 'sweetened beverages':ab,ti OR 'sweetened beverage':ab,ti OR 'beverage, sweetened':ab,ti OR 'beverages, sweetened':ab,ti OR 'sugar-sweetened sodas':ab,ti OR 'sugar sweetened sodas':ab,ti OR 'sugar-sweetened soda':ab,ti OR 'soda, sugar-sweetened':ab,ti OR 'sugar sweetened soda':ab,ti)		
#86.	('gout'/exp OR 'gout':ab,ti OR 'gouts':ab,ti) AND ('coffee'/exp OR 'coffee':ab,ti)	107	21 May 2024
#85.	('gout'/exp OR 'gout':ab,ti OR 'gouts':ab,ti) AND ('tea'/exp OR 'tea':ab,ti OR 'black tea':ab,ti OR 'black teas':ab,ti OR 'tea,black':ab,ti OR 'teas,black':ab,ti OR 'green tea':ab,ti OR 'green teas':ab,ti OR 'tea,green':ab,ti OR 'teas,green':ab,ti)	93	21 May 2024
#84.	'sugar-sweetened beverage'/exp OR 'sugar-sweetened beverages':ab,ti OR 'sugar-sweetened beverage':ab,ti OR 'beverage, sugar-sweetened':ab,ti OR 'beverages, sugar-sweetened':ab,ti OR 'sugar-added beverages':ab,ti OR 'sugar added beverages':ab,ti OR 'sugar sweetened beverage':ab,ti OR 'beverage, sugar sweetened':ab,ti OR 'beverages, sugar sweetened':ab,ti OR 'sugar sweetened beverages':ab,ti OR 'sweetened beverage, sugar':ab,ti OR 'sweetened beverages, sugar':ab,ti OR 'sugar-added beverage':ab,ti OR	8,142	21 May 2024

	'beverages, sugar-added':ab,ti OR 'sugar added beverage':ab,ti OR 'sweetened drinks':ab,ti OR 'sweetened drink':ab,ti OR 'drink, sweetened':ab,ti OR 'drinks, sweetened':ab,ti OR 'sugar-sweetened soft drinks':ab,ti OR 'sugar sweetened soft drinks':ab,ti OR 'sugar-sweetened soft drink':ab,ti OR 'soft drink, sugar-sweetened':ab,ti OR 'soft drinks, sugar-sweetened':ab,ti OR 'sugar sweetened soft drink':ab,ti OR 'sweetened beverages':ab,ti OR 'sweetened beverage':ab,ti OR 'beverage, sweetened':ab,ti OR 'beverages, sweetened':ab,ti OR 'sugar-sweetened sodas':ab,ti OR 'sugar sweetened sodas':ab,ti OR 'sugar-sweetened soda':ab,ti OR 'soda, sugar-sweetened':ab,ti OR 'sugar sweetened soda':ab,ti		
#83.	'coffee'/exp OR 'coffee':ab,ti	27,612	21 May 2024
#82.	'tea'/exp OR 'tea':ab,ti OR 'black tea':ab,ti OR 'black teas':ab,ti OR 'tea,black':ab,ti OR 'teas,black':ab,ti OR 'green tea':ab,ti OR 'green teas':ab,ti OR 'tea,green':ab,ti OR 'teas,green':ab,ti	54,510	21 May 2024
#81.	'osteoporosis'/exp OR 'osteoporosis':ab,ti OR 'osteoporoses':ab,ti OR 'osteoporosis, post-traumatic':ab,ti OR 'osteoporosis, post traumatic':ab,ti OR 'post-traumatic osteoporosis':ab,ti OR 'osteoporosis, senile':ab,ti OR 'senile osteoporoses':ab,ti OR 'senile osteoporosis':ab,ti OR 'osteoporosis, age-related':ab,ti OR 'osteoporosis, age related':ab,ti OR 'bone loss, age-related':ab,ti OR 'age-related bone loss':ab,ti OR 'age-related bone losses':ab,ti OR 'bone loss, age related':ab,ti OR 'age-related osteoporosis':ab,ti OR 'age related osteoporosis':ab,ti OR 'age-related osteoporoses':ab,ti	187,301	21 May 2024
#80.	'rheumatoid arthritis'/exp OR 'rheumatoid arthritis':ab,ti	287,615	21 May 2024
#79.	'osteoarthritis'/exp OR 'osteoarthritis':ab,ti OR 'osteoarthritides':ab,ti OR 'osteoarthrosis':ab,ti OR 'osteoarthroses':ab,ti OR 'arthrosis':ab,ti OR 'arthroses':ab,ti	201,045	21 May 2024
#78.	'gout'/exp OR 'gout':ab,ti OR 'gouts':ab,ti	34,374	21 May 2024

#77. 'sugar sweetened soda':ab,ti	59	21 May 2024
#76. 'soda, sugar-sweetened':ab,ti	3	21 May 2024
#75. 'sugar-sweetened soda':ab,ti	59	21 May 2024
#74. 'sugar sweetened sodas':ab,ti	16	21 May 2024
#73. 'sugar-sweetened sodas':ab,ti	16	21 May 2024
#72. 'beverages, sweetened':ab,ti	72	21 May 2024
#71. 'beverage, sweetened':ab,ti	13	21 May 2024
#70. 'sweetened beverage':ab,ti	2,135	21 May 2024
#69. 'sweetened beverages':ab,ti	5,279	21 May 2024
#68. 'sugar sweetened soft drink':ab,ti	37	21 May 2024
#67. 'soft drinks, sugar-sweetened':ab,ti	11	21 May 2024
#66. 'soft drink, sugar-sweetened':ab,ti	3	21 May 2024
#65. 'sugar-sweetened soft drink':ab,ti	37	21 May 2024
#64. 'sugar sweetened soft drinks':ab,ti	146	21 May 2024
#63. 'sugar-sweetened soft drinks':ab,ti	146	21 May 2024
#62. 'drinks, sweetened':ab,ti	58	21 May 2024
#61. 'drink, sweetened':ab,ti	37	21 May 2024
#60. 'sweetened drink':ab,ti	83	21 May 2024
#59. 'sweetened drinks':ab,ti	475	21 May 2024
#58. 'sugar added beverage':ab,ti	1	21 May 2024
#57. 'beverages, sugar-added':ab,ti	1	21 May 2024
#56. 'sugar-added beverage':ab,ti	1	21 May 2024
#55. 'sweetened beverages, sugar':ab,ti	12	21 May 2024
#54. 'sweetened beverage, sugar':ab,ti	5	21 May 2024
#53. 'sugar sweetened beverages':ab,ti	4,104	21 May 2024
#52. 'beverages, sugar sweetened':ab,ti	24	21 May 2024
#51. 'beverage, sugar sweetened':ab,ti	4	21 May 2024
#50. 'sugar sweetened beverage':ab,ti	1,760	21 May 2024
#49. 'sugar added beverages':ab,ti	15	21 May 2024
#48. 'sugar-added beverages':ab,ti	15	21 May 2024
#47. 'beverages, sugar-sweetened':ab,ti	24	21 May 2024
#46. 'beverage, sugar-sweetened':ab,ti	4	21 May 2024
#45. 'sugar-sweetened beverage':ab,ti	1,761	21 May 2024
#44. 'sugar-sweetened beverages':ab,ti	4,105	21 May 2024
#43. 'sugar-sweetened beverage'/exp	3,643	21 May 2024
#42. 'coffee':ab,ti	24,038	21 May 2024
#41. 'coffee'/exp	18,909	21 May 2024
#40. 'teas,green':ab,ti	32	21 May 2024
#39. 'tea,green':ab,ti	265	21 May 2024
#38. 'green teas':ab,ti	330	21 May 2024
#37. 'green tea':ab,ti	13,396	21 May 2024
#36. 'teas,black':ab,ti	20	21 May 2024
#35. 'tea,black':ab,ti	218	21 May 2024
#34. 'black teas':ab,ti	284	21 May 2024

#33. 'black tea':ab,ti	3,171	21 May 2024
#32. 'tea':ab,ti	49,708	21 May 2024
#31. 'tea'/exp	25,550	21 May 2024
#30. 'age-related osteoporoses':ab,ti	2	21 May 2024
#29. 'age related osteoporosis':ab,ti	349	21 May 2024
#28. 'age-related osteoporosis':ab,ti	349	21 May 2024
#27. 'bone loss, age related':ab,ti	3	21 May 2024
#26. 'age-related bone losses':ab,ti	2	21 May 2024
#25. 'age-related bone loss':ab,ti	1,059	21 May 2024
#24. 'bone loss, age-related':ab,ti	3	21 May 2024
#23. 'osteoporosis, age related':ab,ti	12	21 May 2024
#22. 'osteoporosis, age-related':ab,ti	12	21 May 2024
#21. 'senile osteoporosis':ab,ti	888	21 May 2024
#20. 'senile osteoporoses':ab,ti	3	21 May 2024
#19. 'osteoporosis, senile':ab,ti	11	21 May 2024
#18. 'post-traumatic osteoporosis':ab,ti	17	21 May 2024
#17. 'osteoporosis, post traumatic':ab,ti	1	21 May 2024
#16. 'osteoporosis, post-traumatic':ab,ti	1	21 May 2024
#15. 'osteoporoses':ab,ti	181	21 May 2024
#14. 'osteoporosis':ab,ti	128,640	21 May 2024
#13. 'osteoporosis'/exp	161,938	21 May 2024
#12. 'rheumatoid arthritis':ab,ti	191,748	21 May 2024
#11. 'rheumatoid arthritis'/exp	263,825	21 May 2024
#10. 'arthroses':ab,ti	615	21 May 2024
#9. 'arthrosis':ab,ti	7,943	21 May 2024
#8. 'osteoarthroses':ab,ti	38	21 May 2024
#7. 'osteoarthrosis':ab,ti	4,543	21 May 2024
#6. 'osteoarthritides':ab,ti	5	21 May 2024
#5. 'osteoarthritis':ab,ti	127,303	21 May 2024
#4. 'osteoarthritis'/exp	173,121	21 May 2024
#3. 'gouts':ab,ti	61	21 May 2024
#2. 'gout':ab,ti	23,298	21 May 2024
#1. 'gout'/exp	30,511	21 May 2024

#12. #4 AND #8	159	15 Jan 2025
#11. #3 AND #8	99	15 Jan 2025
#10. #2 AND #8	80	15 Jan 2025
#9. #1 AND #8	225	15 Jan 2025
#8. #5 OR #6 OR #7	39,947	15 Jan 2025
#7. 'beverages, alcoholic':ab,ti	24	15 Jan 2025
#6. 'beverage, alcoholic':ab,ti	2	15 Jan 2025
#5. 'alcoholic beverage'/exp OR 'alcoholic beverage'	39,936	15 Jan 2025
#4. 'osteoporosis'/exp OR 'osteoporosis':ab,ti OR 'osteoporoses':ab,ti OR 'osteoporosis,	193,750	15 Jan 2025

post-traumatic':ab,ti OR 'osteoporosis, post traumatic':ab,ti OR 'post-traumatic osteoporosis':ab,ti OR 'osteoporosis, senile':ab,ti OR 'senile osteoporoses':ab,ti OR 'senile osteoporosis':ab,ti OR 'osteoporosis, age-related':ab,ti OR 'osteoporosis, age related':ab,ti OR 'bone loss, age-related':ab,ti OR 'age-related bone loss':ab,ti OR 'age-related bone losses':ab,ti OR 'bone loss, age related':ab,ti OR 'age-related osteoporosis':ab,ti OR 'age related osteoporosis':ab,ti OR 'age-related osteoporoses':ab,ti

#3.	'rheumatoid arthritis'/exp OR 'rheumatoid arthritis':ab,ti	295,797	15 Jan 2025
#2.	'osteoarthritis'/exp OR 'osteoarthritis':ab,ti OR 'osteoarthritides':ab,ti OR 'osteoarthrosis':ab,ti OR 'osteoarthroses':ab,ti OR 'arthrosis':ab,ti OR 'arthroses':ab,ti	209,138	15 Jan 2025
#1.	'gout'/exp OR 'gout' OR 'gout':ab,ti OR 'gouts':ab,ti	38,552	15 Jan 2025

Web of science

# 检索:

1: gout (主题) OR gouts (主题) and Preprint Citation Index (排除 – 数据库)

2: Osteoarthritis (主题) OR Osteoarthritides (主题) OR Osteoarthrosis (主题) OR Osteoarthroses (主题) OR Arthritis, Degenerative (主题) OR Arthritides, Degenerative (主题) OR Degenerative Arthritides (主题) OR Degenerative Arthritis (主题) OR Arthrosis (主题) OR Arthroses (主题) OR Osteoarthrosis Deformans (主题) and Preprint Citation Index (排除 – 数据库) 检索结果: 195324

3: Rheumatoid Arthritis (主题) and Preprint Citation Index (排除 – 数据库)

4: Osteoporosis (主题) OR Osteoporoses (主题) OR Osteoporosis, Post-Traumatic (主题) OR Osteoporosis, Post Traumatic (主题) OR Post-Traumatic Osteoporoses (主题) OR Post-Traumatic Osteoporosis (主题) OR Osteoporosis, Senile (主题) OR Osteoporoses, Senile (主题) OR Senile Osteoporoses (主题) OR Osteoporosis, Involutional (主题) OR Senile Osteoporosis (主题) OR Osteoporosis, Age-Related (主题) OR Osteoporosis, Age Related (主题) OR Bone Loss, Age-Related (主题) OR Age-Related Bone Loss (主题) OR Age-Related Bone Losses (主题) OR Bone Loss, Age Related (主题) OR Bone Losses, Age-Related (主题) OR Age-Related Osteoporosis (主题) OR Age Related Osteoporosis (主题) OR Age-Related Osteoporoses (主题) OR Osteoporoses, Age-Related (主题) and Preprint Citation Index (排除 – 数据库)检索结果: 195216

5: tea (主题) OR black tea (主题) OR black teas (主题) OR tea，black (主题) OR teas，black (主题) OR green tea (主题) OR green teas (主题) OR tea，green (主题) OR teas，green (主题) and Preprint Citation Index (排除 – 数据库) 运行日期: Tue May 21 2024 17:30:59 GMT+0800 (中国标准时间) 检索结果: 103268

6: coffee (主题) and Preprint Citation Index (排除 – 数据库) 检索结果: 57550

7: Sugar-Sweetened Beverages (主题) OR Beverage, Sugar-Sweetened (主题) OR Beverages, Sugar-Sweetened (主题) OR Sugar-Sweetened Beverage (主题) OR Sugar-Added Beverages (主题) OR Sugar Added Beverages (主题) OR Sugar Sweetened Beverage (主题) OR Beverage, Sugar Sweetened (主题) OR Beverages, Sugar Sweetened (主题) OR Sugar Sweetened Beverages (主题) OR Sweetened Beverage, Sugar (主题) OR Sweetened Beverages, Sugar (主题) OR Sugar-Added Beverage (主题) OR Beverage, Sugar-Added (主题) OR Beverages, Sugar-Added (主题) OR Sugar Added Beverage (主题) OR Sweetened Drinks (主题) OR Sweetened Drink (主题) OR Drink, Sweetened (主题) OR Drinks, Sweetened (主题) OR Sugar-Sweetened Soft Drinks (主题) OR Sugar Sweetened Soft Drinks (主题) OR Sugar-Sweetened Soft Drink (主题) OR Drink, Sugar-Sweetened Soft (主题) OR Drinks, Sugar-Sweetened Soft (主题) OR Soft Drink, Sugar-Sweetened (主题) OR Soft Drinks, Sugar-Sweetened (主题) OR Sugar Sweetened Soft Drink (主题) OR Sweetened Beverages (主题) OR Sweetened Beverage (主题) OR Beverage, Sweetened (主题) OR Beverages, Sweetened (主题) OR Sugar-Sweetened Sodas (主题) OR Sugar Sweetened Sodas (主题) OR Sugar-Sweetened Soda (主题) OR Soda, Sugar-Sweetened (主题) OR Sodas, Sugar-Sweetened (主题) OR Sugar Sweetened Soda (主题) and Preprint Citation Index (排除 – 数据库) 检索结果: 15678

8: #1 AND #5 and Preprint Citation Index (排除 – 数据库) 检索结果: 99

9: #1 AND #6 and Preprint Citation Index (排除 – 数据库) 检索结果: 130

10: #1 AND #7 and Preprint Citation Index (排除 – 数据库) 检索结果: 104

11: #2 AND #5 and Preprint Citation Index (排除 – 数据库) 检索结果: 287

12: #2 AND #6 and Preprint Citation Index (排除 – 数据库) 检索结果: 45

13: #2 AND #7 and Preprint Citation Index (排除 – 数据库) 检索结果: 10

14: #3 AND #5 and Preprint Citation Index (排除 – 数据库) 检索结果: 406

15: #3 AND #6 and Preprint Citation Index (排除 – 数据库) 检索结果: 140

16: #3 AND #7 and Preprint Citation Index (排除 – 数据库) 检索结果: 19

17: #4 AND #5 and Preprint Citation Index (排除 – 数据库) 检索结果: 478

18: #4 AND #6 and Preprint Citation Index (排除 – 数据库) 检索结果: 278

19: #4 AND #7 and Preprint Citation Index (排除 – 数据库) 检索结果: 32

20: #8 OR #9 OR #10 OR #11 OR #12 OR #13 OR #14 OR #15 OR #16 OR #17 OR #18 OR #19 and Preprint Citation Index 检索结果: 1693

1: (((TS=(Beverage, Alcoholic)) OR TS=(Alcoholic Beverages)) OR TS=(Alcoholic Beverage)) OR TS=(Beverages, Alcoholic)

and Preprint Citation Index (排除 – 数据库) 运行日期: Wed Jan 15 2025 14:40:21 GMT+0800 (中国标准时间) 检索结果: 89198

2: (TS=(gout)) OR TS=(gouts) and Preprint Citation Index (排除 – 数据库) 运行日期: Wed Jan 15 2025 14:42:33 GMT+0800 (中国标准时间) 检索结果: 43887

3: (((((((((TS=(Osteoarthritis)) OR TS=(Osteoarthritides)) OR TS=(Osteoarthrosis)) OR TS=(Osteoarthroses)) OR TS=(Arthritis, Degenerative)) OR TS=(Arthritides, Degenerative)) OR TS=(Degenerative Arthritides)) OR TS=(Degenerative Arthritis)) OR TS=(Arthrosis)) OR TS=(Arthroses)) OR TS=(Osteoarthrosis Deformans) and Preprint Citation Index (排除 – 数据库) 运行日期: Wed Jan 15 2025 14:44:28 GMT+0800 (中国标准时间) 检索结果: 237427

4: TS=(Rheumatoid Arthritis) and Preprint Citation Index (排除 – 数据库) 运行日期: Wed Jan 15 2025 14:44:55 GMT+0800 (中国标准时间) 检索结果: 366240

5: ((((((((((((((((((((TS=(Osteoporosis)) OR TS=(Osteoporoses)) OR TS=(Osteoporosis, Post-Traumatic)) OR TS=(Osteoporosis, Post Traumatic)) OR TS=(Post-Traumatic Osteoporoses)) OR TS=(Post-Traumatic Osteoporosis)) OR TS=(Osteoporosis, Senile)) OR TS=(Osteoporoses, Senile)) OR TS=(Senile Osteoporoses)) OR TS=(Osteoporosis, Involutional)) OR TS=(Senile Osteoporosis)) OR TS=(Osteoporosis, Age-Related)) OR TS=(Osteoporosis, Age Related)) OR TS=(Bone Loss, Age-Related)) OR TS=(Age-Related Bone Loss)) OR TS=(Age-Related Bone Losses)) OR TS=(Bone Loss, Age Related)) OR TS=(Bone Losses, Age-Related)) OR TS=(Age-Related Osteoporosis)) OR TS=(Age Related Osteoporosis)) OR TS=(Age-Related Osteoporoses)) OR TS=(Osteoporoses, Age-Related)) NOT (SILOID==("PPRN")) 运行日期: Wed Jan 15 2025 14:48:00 GMT+0800 (中国标准时间) 检索结果: 235242

6: #1 AND #2 and Preprint Citation Index (排除 – 数据库) 运行日期: Wed Jan 15 2025 14:48:13 GMT+0800 (中国标准时间) 检索结果: 188

7: #1 AND #3 and Preprint Citation Index (排除 – 数据库) 运行日期: Wed Jan 15 2025 14:48:22 GMT+0800 (中国标准时间) 检索结果: 92

8: #1 AND #4 and Preprint Citation Index (排除 – 数据库) 运行日期: Wed Jan 15 2025 14:48:30 GMT+0800 (中国标准时间) 检索结果: 165

9: #1 AND #5 and Preprint Citation Index (排除 – 数据库) 运行日期: Wed Jan 15 2025 14:49:45 GMT+0800 (中国标准时间) 检索结果: 413

1. Chi, X., et al., *Effects of dietary factors on hyperuricaemia and gout: a systematic review and meta-analysis of observational studies.* International Journal of Food Sciences and Nutrition, 2024. **75**(8): p. 753-773.

Table S2：The studies we excluded

[1]	Lack of available data
**[2]**	Lack of available data
**[3]**	Lack of available data
**[4]**	Lack of available data
**[5]**	Lack of available data
**[6]**	Lack of available data
**[7]**	Lack of available data
**[8]**	Lack of available data
**[9]**	Umbrella review
**[10]**	Meta-analysis of Mendelian randomization

References：

1. Hamel, C., et al., *Do sugar-sweetened beverages cause adverse health outcomes in adults? A systematic review protocol.* Syst Rev, 2014. **3**: p. 108.
2. Moi, J.H., et al., *Lifestyle interventions for chronic gout.* Cochrane Database Syst Rev, 2013. **2013**(5): p. Cd010039.
3. Moi, J.H., et al., *Lifestyle interventions for acute gout.* Cochrane Database Syst Rev, 2013. **2013**(11): p. Cd010519.
4. Asoudeh, F., et al., *A systematic review and meta-analysis of prospective cohort studies on the association between alcohol intake and risk of fracture.* Critical Reviews in Food Science and Nutrition, 2022. **62**(20): p. 5623-5637.
5. B., N.-I., *The role of alcohol consumption in pathogenesis of gout.* Critical Reviews in Food Science and Nutrition, 2022. **62**(25): p. 7129-7137.
6. To, K., et al., *The association between alcohol consumption and osteoarthritis: a meta-analysis and meta-regression of observational studies.* Rheumatology International, 2021. **41**(9): p. 1577-1591.
7. Berg KM, K.H., Jackson JL, et al., *Association between alcohol consumption and both osteoporotic fracture and bone density.* Am J Med, 2008. **121**(5): p. 406-18.
8. Chi X, C.Y., Yang B, et al., *Effects of dietary factors on hyperuricaemia and gout: a systematic review and meta-analysis of observational studies.* International Journal of Food Sciences and Nutrition, 2024. **75**(8): p. 753-773.
9. Feng, W., et al., *Role of diet in osteoporosis incidence: Umbrella review of meta-analyses of prospective observational studies.* Crit Rev Food Sci Nutr, 2023. **63**(19): p. 3420-3429.
10. Wang, J., et al., *The Causal Association between Alcohol, Smoking, Coffee Consumption, and the Risk of Arthritis: A Meta-Analysis of Mendelian Randomization Studies.* Nutrients, 2023. **15**(23).

Table S3: Quality evaluation of included studies.

study	1	2	3	4	5	6	7	8	9	10	11	12	13	14	15	16	Overall rating	All
Zhang ZF (10)	Y	Y	Y	Y	Y	Y	N	Y	Y	N	Y	Y	Y	Y	Y	N	13	low
Ayoub-Charette, S (39)	Y	Y	Y	Y	Y	N	Y	Y	Y	N	Y	Y	Y	Y	Y	N	13	moderate
Ayoub-Charette S (12)	Y	Y	Y	Y	Y	N	Y	Y	Y	Y	Y	N	N	Y	N	Y	12	low
Ebrahimpour-Koujan S (13)	Y	N	Y	Y	Y	Y	Y	Y	Y	Y	Y	Y	Y	Y	Y	Y	15	low
Zhang Y (36)	Y	N	Y	Y	Y	Y	Y	Y	Y	Y	Y	N	N	Y	Y	Y	13	low
Chen CC (28)	Y	Y	Y	Y	Y	N	Y	Y	Y	Y	Y	Y	N	Y	Y	Y	14	moderate
Lee YH (42)	Y	N	Y	Y	N	N	N	Y	N	Y	Y	N	N	Y	Y	Y	9	very low
Park KY (37)	Y	N	Y	Y	Y	Y	Y	Y	Y	N	Y	Y	N	Y	Y	N	12	low
Zeng X (32)	Y	Y	Y	Y	N	N	Y	Y	Y	Y	Y	Y	Y	N	Y	Y	13	moderate
Asoudeh F (41)	Y	Y	Y	Y	Y	Y	Y	Y	Y	N	Y	Y	N	N	Y	N	12	moderate
Long Z (43)	Y	Y	Y	Y	N	Y	Y	Y	Y	N	Y	Y	Y	Y	Y	Y	14	moderate
Guo M (29)	Y	Y	Y	Y	Y	Y	Y	Y	Y	N	Y	Y	Y	Y	Y	N	14	moderate
Zhou F (31)	Y	Y	Y	Y	Y	Y	Y	Y	Y	Y	Y	Y	Y	Y	Y	Y	16	high
Zhang Y (40)	Y	N	Y	Y	Y	Y	Y	Y	Y	Y	Y	Y	Y	Y	Y	Y	15	low
Sun K (30)	Y	N	Y	N	N	N	Y	Y	Y	N	Y	Y	N	Y	Y	Y	10	very low
Li R (38)	Y	N	Y	Y	Y	Y	Y	Y	Y	N	Y	Y	Y	Y	Y	Y	14	low
Ke Y (34)	Y	Y	Y	Y	Y	Y	Y	Y	Y	N	Y	Y	Y	Y	Y	Y	15	high
Wieczorek M (44)	Y	Y	Y	Y	Y	Y	N	Y	Y	N	Y	Y	Y	Y	Y	Y	14	low
Cheraghi Z (35)	Y	N	Y	Y	Y	Y	N	Y	Y	N	Y	Y	Y	Y	Y	Y	12	very low
Li W (33)	Y	Y	Y	Y	Y	Y	Y	Y	Y	Y	Y	Y	Y	N	N	Y	14	moderate

Scale of item score: N, no; Y, yes.

The AMSTAR criteria are Q1: Did the research questions and inclusion criteria for the review include the components of PICO?, Q2: Did the report of the review contain an explicit statement that the review methods

were established prior to the conduct of the review and did the report justify any significant deviations from the protocol?, Q3: Did the review authors explain their selection of the study designs for inclusion in the review?, Q4: Did the review authors use a comprehensive literature search strategy?, Q5: Did the review authors perform study selection in duplicate?, Q6: Did the review authors perform data extraction in duplicate?, Q7: Did the review authors provide a list of excluded studies and justify the exclusions?, Q8: Did the review authors describe the included studies in adequate detail?, Q9: Did the review authors use a satisfactory technique for assessing the risk of bias (RoB) in individual studies that were included in the review?, Q10: Did the review authors report on the sources of funding for the studies included in the review?, Q11: If meta-analysis was performed, did the review authors use appropriate methods for statistical combination of results?, Q12: If meta-analysis was performed, did the review authors assess the potential impact of RoB in individual studies on the results of the meta-analysis or other evidence synthesis?, Q13: Did the review authors account for RoB in primary studies when interpreting/discussing the results of the review?, Q14: Did the review authors provide a satisfactory explanation for, and discussion of, any heterogeneity observed in the results of the review?, Q15: If they performed quantitative synthesis did the review authors carry out an adequate investigation of publication bias (small study bias) and discuss its likely impact on the results of the review?, Q16: Did the review authors report any potential sources of conflict of interest, including any funding they received for conducting the review?

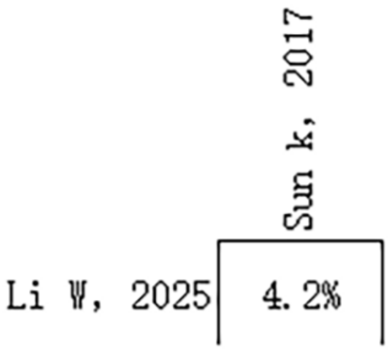

Figure S1: The GROOVE of the incidence of osteoporosis by drinking tea with OR as statistic metric

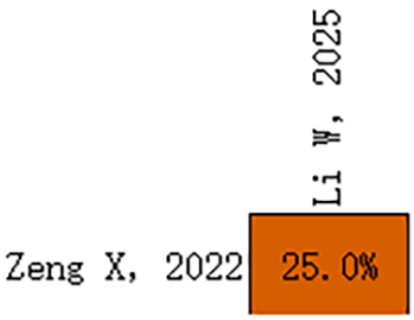

Figure S2: The GROOVE of the incidence of osteoporosis by drinking coffee with OR as statistic metric

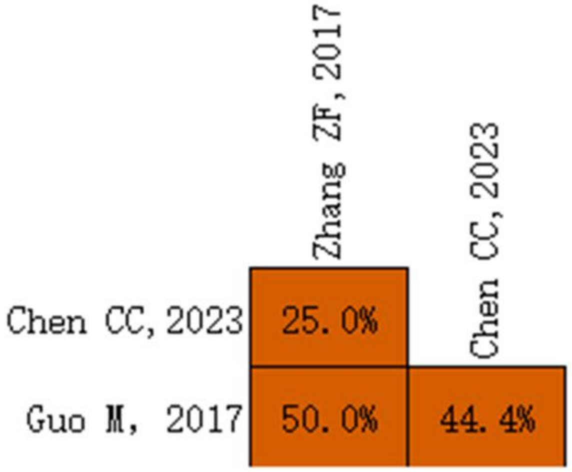

Figure S3: The GROOVE of bone mineral density (BMD) value by drinking tea with MD as statistic metric

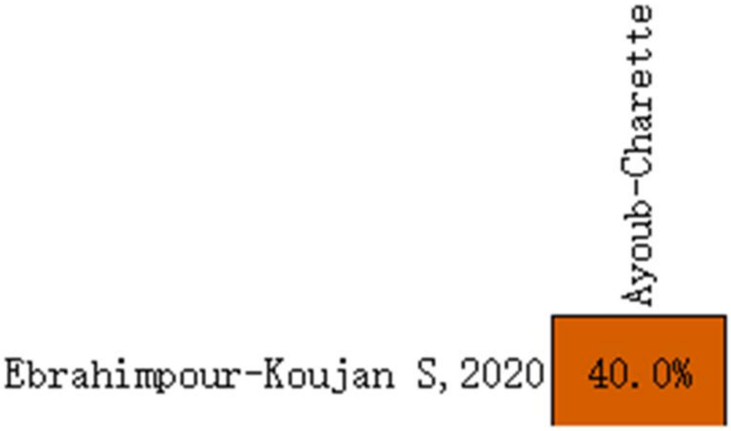

Figure S4: The GROOVE of the incidence of gout by drinking SSBs with RR as statistic metric

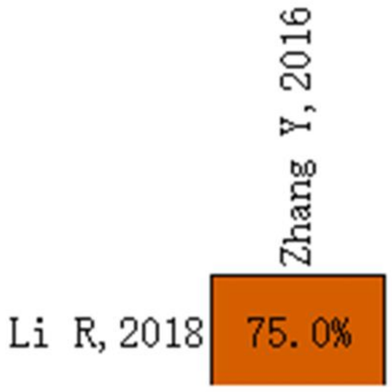

Figure S5: The GROOVE of the incidence of hyperuricemia (HUA) by drinking coffee with OR as statistic metric

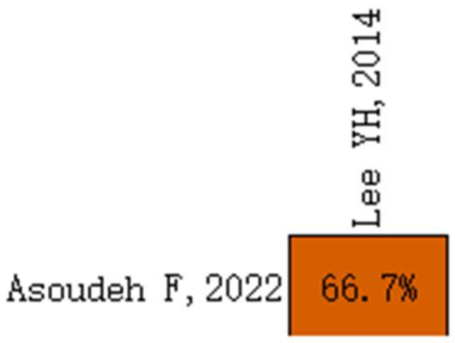

Figure S6: The GROOVE of the incidence of rheumatoid arthritis by drinking coffee with RR as statistic metric

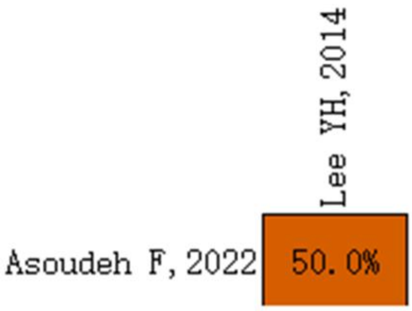

Figure S7: The GROOVE of the incidence of rheumatoid arthritis by drinking tea with RR as statistic metric

Table S4: The GRADE of the incidence of osteoporosis

Author	intervention	Type of metric	No. of Studies	Overall Certainty (GRADE)	Risk of Bias	Inconsistency	Indirectness	Imprecision	Publication Bias
Zhou F(31)	tea	RR	5	Very Low	Serious	Not Serious	Not Serious	Serious	Serious
Sun K(30)	tea	OR	17	Very Low	Serious	Serious	Not Serious	Serious	Serious
Li W(33)	tea	OR	8	Low	Serious	Not Serious	Not Serious	Not Serious	Serious
Li W(33)	coffee	OR	11	Low	Serious	Not Serious	Not Serious	Not serious	Serious
Zeng X(32)	coffee	OR	4	Low	Serious	Not Serious	Not Serious	Serious	Possible
Cheraghi Z (35)	alcohol	OR	6	Very Low	Serious	Not Serious	Not Serious	Serious	Serious

Table S5: The GRADE of Bone mineral density (BMD) value

Author	intervention	Type of metric	No. of Studies	Overall Certainty (GRADE)	Risk of Bias	Inconsistency	Indirectness	Imprecision	Publication Bias
Zhang ZF(10)	tea	OR	5	Low	Serious	Not Serious	Not Serious	Not Serious	Serious
Zhang ZF(10)	tea	MD	2	Very Low	Serious	Not Serious	Not Serious	Serious	Serious
Chen CC(28)	tea	MD	7	Very Low	Serious	Serious	Not Serious	Serious	Serious
Guo M(29)	tea	MD	4	Very Low	Serious	Serious	Not Serious	Serious	Serious
Guo M(29)	tea	MD	3	Very Low	Serious	Not Serious	Not Serious	Serious	Serious
Zhou F(31)	tea	SMD	10	Very Low	Serious	Not Serious	Not Serious	Serious	Serious
Chen CC(28)	coffee	MD	3	Very Low	Serious	Not Serious	Not Serious	Serious	Serious

Table S6: The GRADE of the value of Serum uric acid (SUA)

Author	intervention	Type of metric	No. of Studies	Overall Certainty (GRADE)	Risk of Bias	Inconsistency	Indirectness	Imprecision	Publication Bias
Ayoub-Charette S(39)	SSB	MD	9	High	Not Serious	Not Serious	Not Serious	Not Serious	Not Serious
Ayoub-Charette S(39)	SSB	MD	13	High	Not Serious	Not Serious	Not Serious	Not Serious	Not Serious
Ayoub-Charette S(39)	SSB	MD	4	Moderate	Not Serious	Not Serious	Not Serious	Not Serious	Serious
Zhang Y(36)	coffee	SMD	6	Low	Serious	Not Serious	Not Serious	Not Serious	Serious
Park KY(37)	coffee	MD	7	Very Low	Serious	Not Serious	Not Serious	Serious	Serious
Zhang Y(40)	tea	WMD	9	Very Low	Serious	Not serious	Not Serious	Serious	Serious

Table S7: The GRADE of the incidence of gout

Author	intervention	Type of metric	No. of Studies	Overall Certainty (GRADE)	Risk of Bias	Inconsistency	Indirectness	Imprecision	Publication Bias
Ayoub-Charette S(12)	SSB	RR	2	Moderate	Not Serious	Not Serious	Not Serious	Not Serious	Serious
Ebrahimpour-Koujan S(13)	SSB	RR	3	Very Low	Serious	Not Serious	Not Serious	Serious	Serious
Ebrahimpour-Koujan S(13)	SSB	RR	2	Very Low	Serious	Not Serious	Serious	Serious	Serious
Li R(38)	SSB	OR	2	Low	Serious	Not Serious	Not serious	Not Serious	Serious

Zhang Y(36)	coffee	RR	2	Very Low	Serious	Not Serious	Not Serious	Serious	Serious
Li R(38)	coffee	OR	2	Very Low	Serious	Not Serious	Not Serious	Serious	Serious

Table S8: The GRADE of the incidence of hyperuricemia (HUA)

Author	intervention	Type of metric	No. of Studies	Overall Certainty (GRADE)	Risk of Bias	Inconsistency	Indirectness	Imprecision	Publication Bias
Ebrahimpour-Koujan S(13)	SSB	RR	4	Low	Serious	Not Serious	Not Serious	Not Serious	Serious
Li R(38)	SSB	OR	4	Moderate	Not Serious	Not Serious	Not Serious	Not Serious	Serious
Zhang Y(36)	coffee	OR	4	Low	Serious	Not Serious	Not Serious	Not Serious	Serious
Li R(38)	coffee	OR	3	Very Low	Serious	Not Serious	Not Serious	Serious	Serious
Zhang Y(40)	tea	OR	6	Very Low	Serious	Not Serious	Serious	Serious	Serious
Ebrahimpour-Koujan S(13)	SSB	RR	4	Low	Serious	Not Serious	Not Serious	Not Serious	Serious

Table S9: The GRADE of the incidence of rheumatoid arthritis

Author	intervention	Type of metric	No. of Studies	Overall Certainty (GRADE)	Risk of Bias	Inconsistency	Indirectness	Imprecision	Publication Bias
Lee YH(42)	coffee	RR	5	Low	Serious	Not Serious	Not Serious	Not Serious	Serious
Asoudeh F(41)	coffee	RR	5	Low	Serious	Not Serious	Not Serious	Not Serious	Serious
Lee YH(42)	tea	RR	3	Very Low	Serious	Not Serious	Serious	Serious	Serious
Asoudeh F(41)	tea	RR	3	Low	Serious	Not Serious	Not Serious	Not Serious	Serious

Table S10: The results table of incidence of osteoporosis

Author	Year	intervention	Number of studies included	Type of metric	Effect	95%CI	Effects model	I^2	P	GRADE	Evidence class
Zhou F[31]	2024	tea	5	RR	0.8	0.674 to 0.950	random	82	<0.05	very low	IV
Sun K[30]	2017	tea	17	OR	0.62	0.46 to 0.83	random	94	0.002	very low	IV
Li W[33]	2025	tea	8	OR	0.75	0.62 to 0.91	random	80.4	< 0.0001	low	III
Li W[33]	2025	coffee	11	OR	0.79	0.73 to 0.84	fixed	28.9	0.17	low	NS
Zeng X[32]	2022	coffee	4	OR	0.79	0.65 to 0.92	fixed	23.3	<0.05	low	IV
Cheraghi Z [35]	2019	alcohol	6	OR	2.95	1.78 to 4.9	random	0	<0.0001	low	III

Table S11: The results table of BMD value

Author	Year	intervention	Number of studies included	Type of metric	Effect	95%CI	Effects model	I^2	P	GRADE	Evidence class
Zhang ZF[10]	2017	tea	5	OR	0.66	0.47 to 0.94	random	46	0.02	low	IV
Zhang ZF[10]	2017	tea	2	MD	0	0 to 0	random	0	0.06	very low	NS
Chen CC[28]	2023	tea	7	MD	0.039	0.012 to 0.09	random	98.455	0.132	very low	NS
Guo M[29]	2017	tea	4	MD	0.04	0.01 to 0.08	random	88	0.02	very low	IV

Guo M[29]	2017	tea	3	MD	0.01	0.01 to 0.01	random	0	<0.01	very low	I
Zhou F[31]	2024	tea	10	SMD	0.332	0.207 to 0.457	random	94	<0.05	very low	IV
Chen CC[28]	2023	coffee	3	MD	0.02	-0.003 to 0.044	random	0	0.093	very low	NS

Table S12: The results table of the value of SUA

Author	Year	intervention	Number of studies included	Type of metric	Effect	95%CI	Effects model	I^2	P	GRADE	Evidence class
Ayoub-Charette S[39]	2021	SSB	9	MD	0.42	0.24 to 0.59	random	33	<0.01	high	IV
Ayoub-Charette S[39]	2021	SSB	13	MD	0.43	0.23 to 0.63	random	56.17	<0.01	high	IV
Ayoub-Charette S[39]	2021	SSB	4	MD	0.09	-0.14 to 0.32	fixed	0	0.45	moderate	NS
Zhang Y[36]	2016	coffee	6	SMD	-0.09	-0.23 to 0.05	random	84.2	0.21	low	NS
Park KY[37]	2016	coffee	7	MD	-0.09	-0.43 to 0.25	random	97	0.61	very low	NS
Zhang Y[36]	2017	tea	9	WMD	7.41	-2.34 to 17.15	random	92.5	0.136	very low	NS

Table S13: The results table of the incidence of gout

Author	Year	intervention	Number of studies included	Type of metric	Effect	95%CI	Effects model	I^2	P	GRADE	Evidence class
Ayoub-Charette S[12]	2019	SSB	2	RR	2.08	1.40 to 3.08	random	0	0.0003	moderate	III
Ebrahimpour-Koujan S[13]	2020	SSB	3	RR	1.35	1.18 to 1.55	random	40.1	<0.05	very low	IV
Ebrahimpour-Koujan S[13]	2020	SSB	2	RR	1.33	1.06 to 1.66	random	0	<0.05	very low	IV
Li R[38]	2018	SSB	2	OR	2.14	1.65 to 2.78	fixed	0	<0.01	low	I
Zhang Y[40]	2016	coffee	2	RR	0.43	0.31 to 0.59	fixed	0	<0.001	very low	III
Li R[38]	2018	coffee	2	OR	0.47	0.37 to 0.59	random	39	<0.01	very low	I

Table S14: The results table of the incidence of hyperuricemia (HUA)

Author	Year	intervention	Number of studies included	Type of metric	Effect	95%CI	Effects model	I^2	P	GRADE	Evidence class
Ebrahimpour-Koujan S[13]	2020	SSB	4	RR	1.35	1.19 to 1.52	random	41.4	<0.05	low	IV
Li R[38]	2018	SSB	4	OR	1.85	1.66 to 2.07	fixed	31	<0.01	moderate	I
Zhang Y[40]	2016	coffee	4	OR	0.84	0.65 to 1.09	fixed	22.6	0.2	low	NS
Li R[38]	2018	coffee	3	OR	0.96	0.76 to 1.22	random	52	0.75	very low	NS

Zhang Y[36]	2017	tea	6	OR	0.98	0.77 to 1.24	random	72.3	0.839	very low	NS

Table S15: The results table of the incidence of rheumatoid arthritis

Author	Year	intervention	Number of studies included	Type of metric	Effect	95%CI	Effects model	I^2	P	GRADE	Evidence class
Lee YH[42]	2014	coffee	5	RR	2.426	1.060 to 5.554	random	89	0.036	low	IV
Asoudeh F[41]	2022	coffee	5	RR	1.3	1.04 to 1.62	fixed	0	<0.05	low	IV
Lee YH[42]	2014	tea	3	RR	0.88	0.624 to 1.239	fixed	44.2	0.463	very low	NS
Asoudeh F[41]	2022	tea	3	RR	1.05	0.73 to 1.53	fixed	69.1	>0.05	low	NS
